# Exploration of novel ecological niches for pigmented bacteria with dye bioremediation potential

**DOI:** 10.3389/fmicb.2026.1895442

**Published:** 2026-10-01

**Authors:** Vasundhara More, Rucha Bagade, Vaishnavi Mokashi, Shilpa Mujumdar, Ujwala Khisti

**Affiliations:** 1 Modern College of Arts, Science and Commerce (Autonomous), Pune, India; 2 Annasaheb Magar Mahavidyalaya, Pune, India

**Keywords:** azo dye, bioremediation, dye degradation, microbial pigments, niche, pigmented bacteria, synthetic dyes, triphenylmethane dye

## Abstract

**Introduction:**

This study explores the comparative bioremediation potential of pigmented bacteria isolated from diverse ecological niches. Three distinct isolates were identified: *Shewanella xiamenensis* (U2f, from goldfish gills), *Paracoccus* sp. (THN1, a rose thorn endophyte), and *Kocuria* sp. (YC, an incidental laboratory isolate).

**Methods:**

The isolated bacterial pigments were characterized using FTIR spectroscopy. The isolates and a developed bacterial consortium were evaluated for their ability to tolerate, decolorize, and degrade various synthetic dyes (Congo red, malachite green, bromophenol blue, methylene blue, and crystal violet). The resulting decolorization and biodegradation processes were validated using UV–Vis spectroscopic analysis, FTIR spectroscopy, and HPLC.

**Results:**

FTIR analysis indicated that the tan pigment from *S. xiamenensis* (U2f) was similar to melanin, the bright orange pigment from *Paracoccus* sp. (THN1) to astaxanthin, and the yellow pigment from *Kocuria* sp. (YC) to carotenoids. For bioremediation, *S. xiamenensis* (U2f) successfully decolorized the azo dye Congo red up to 200 mg/mL (70% efficiency) and tolerated levels up to 400 mg/mL. Conversely, *Paracoccus* sp. (THN1) and *Kocuria* sp. (YC) decolorized the triphenylmethane dye malachite green up to 200 mg/mL with 80–91% efficiency, tolerating concentrations up to 500 mg/mL. Notably, a consortium of *Paracoccus* sp. and *Kocuria* sp. achieved 100% degradation efficiency of malachite green (200 mg/mL) within 16 hours under shaking conditions.

**Discussion:**

These findings highlight the unique, niche-specific bioremediation capabilities of pigmented bacteria. The results demonstrate that targeted single isolates or designed consortia exhibit distinct and highly efficient degradation pathways against specific classes of toxic synthetic dyes, offering a promising tool for targeted environmental cleanup.

## 1 Introduction

The rapid expansion of the global industrial sector, particularly in industries such as textiles, paper, leather, and cosmetics, has led to a significant increase in the discharge of synthetic dyes into the environment. Annually, approximately 7 × 10^7^ tons of synthetic dyes are produced worldwide, with over 10,000 tons generated solely by the textile industry (Zayed et al., 2023). As these compounds persist in water bodies, they obstruct sunlight penetration, disrupt photosynthetic activity, and induce toxicity due to the presence of heavy metals and aromatic amines (Akansha et al., 2022; Desai et al., 2021). Due to their complex structures and inherent resistance, these dyes pose significant health and environmental hazards (Al-Fayaad and Khudhair, 2022; BERA et al., 2022). In textile industries, during the coloring process, these dyes exhibit poor binding to fabrics, resulting in a significant amount of dye being discharged as untreated effluent into aquatic environments such as rivers, lakes, and ponds (Subburaj et al., 2024). These effluents pose a great threat to ecosystems by increasing the biochemical oxygen demand (BOD) and chemical oxygen demand (COD), thereby altering the physicochemical parameters of the receiving water bodies and creating conditions that impede the survival of aquatic life while promoting the bioaccumulation of toxic compounds (Olukanni et al., 2019). The high solubility and synthetic origin of these textile dyes, along with their carcinogenic and mutagenic effects on humans and animals, highlight the urgent need for effective treatment strategies (Tanaya et al., 2024). There are three categories of synthetic dyes used in the textile industry, including azo, anthraquinone, and triphenylmethane dyes (Akansha et al., 2022; Kusumlata et al., 2024). Among these, the azo group (Procion red, methylene blue, Congo red, indigo carmine, etc.) dominates the market, accounting for approximately 70% of total dye production, while triphenylmethane (malachite green, crystal violet, basic fuchsin, and brilliant green), anthraquinone (alizarin, disperse blue 1, reactive blue 19, and acid blue 25), and other variants constitute the remaining share of dye production (Birmole and Samudravijay, 2022). These azo dyes are synthetic organic dyes with the functional group R–N=N–R’, in which R and R′ are mainly aryl groups, with C–N=N–C linkages (Das et al., 2023). Despite their extensive usage, a significant proportion of synthetic dyes remain unfixed during processing and are released into water bodies as toxic effluent (Kusumlata et al., 2024; Moyo et al., 2022). Traditional treatment approaches, such as physical and chemical techniques involving processes like flocculation, coagulation, filtration, adsorption, precipitation, oxidation, ion exchange, and membrane separation, often fail due to high operational costs, low stability and durability, excessive sludge generation, and the potential for secondary pollution (Chandran et al., 2025; El-sheshtawy and Abdelkreem, 2020). In response to this, microbiological remediation strategies have emerged as a potential alternative (Akansha et al., 2022). Bioremediation has emerged as a promising technology that utilizes microorganisms’ potential to degrade environmental pollutants. Among these biological agents, pigmented bacteria have gained attention for their ability to enzymatically degrade complex azo and synthetic dyes and use them as a carbon source to generate energy, thereby reducing the dye concentration in textile effluents and converting them into less toxic intermediates (BERA et al., 2022; Kusumlata et al., 2024; Madhuri et al., 2018). The enzymatic machinery of these bacteria primarily involves oxidoreductase, such as azo-reductases, laccases, and peroxidases, which catalyze the cleavage of toxic dye compounds (Das et al., 2023; Abd El-Rahim et al., 2025). These enzymatic degradation capabilities offer a notable advantage over conventional treatment methods, which often generate large quantities of toxic sludge and may fail to completely eliminate contaminants. Among the various microbial candidates, pigmented bacteria are of particular interest due to their ability to produce bioactive secondary metabolites such as prodigiosin, melanin, carotenoids, and violacein that are synthesized primarily in response to impaired metabolism or stressful environmental conditions (Narsing Rao et al., 2017; Agarwal et al., 2023). Consequently, these metabolites contribute to the survival of the organisms under extreme conditions by offering protection against oxidative stress and radiation, as well as by enhancing their capability to degrade complex dye structures in contaminated effluents (Gomes and Soares, 2023; Rathour et al., 2024). Pigmented bacterial strains possess unique enzymatic pathways that enable them to tolerate and detoxify the toxic effects associated with high dye concentrations and complex effluent composition (Rathour et al., 2024). These properties make pigmented bacteria promising candidates for future bioremediation applications, particularly as industries shift toward sustainable alternatives that minimize hazardous waste and environmental toxicity (Anshi et al., 2024; Tiwari, 2022). Exploring the microorganisms from different ecological niches, such as soil, aquatic environments, and the rhizosphere, that produce pigments can provide critical insight into their specific degradation capabilities (Barreto et al., 2023).

Ecologically diverse and stress-prone niches can harbor pigmented bacteria with enhanced metabolic diversity, including the ability to decolorize and degrade dyes. Rose prickles/thorns are outgrowths of the epidermal layer or tissue; in terms of chemical composition, prickles are composed of polymers of cellulose, hemicellulose, lignin, mineral substances, polymerized organic compounds, suberin, etc. (Baroni et al., 2012). They form an impermeable barrier that restricts the free movement of nutrients, leading to the starvation of pathogens. Their insoluble, rigid, and indigestible nature makes them key components of an excellent physical barrier (Doughari, 2015). Prickles are the first line of defense in plants against different biotic and abiotic stresses and play a particularly important role in herbivore defense (Zhou et al., 2021). Rose thorns or prickles represents a harsh microenvironment where pigmentation may act as a protective barrier. Plant-associated bacteria, such as epiphytes and endophytes are exposed to different oxidative and chemical stresses. These bacteria are equipped with different enzymatic systems, such as laccases and peroxidases that have detoxification capabilities (Das et al., 2023). The ecological niche of fish is known to be a rich source of microbial diversity. Bacteria isolated from this environment are known to possess unique and varied metabolic capabilities. However, fish gills are constantly exposed to dissolved organic matter, pollutants, and variable oxygen levels within the aquatic habitat. Therefore, gills harbor microbes adapted to biofilm formation and xenobiotic interaction, and pigmentation may help provide protection against ROS in aquatic environment (Pratte et al., 2018). Aquatic microbiota, especially gill-associated communities, are adapted to pollution-rich environments, making them potential candidates for dye biodegradation. Hence, these niches, i.e., terrestrial and aquatic environments, represent distinct ecological stress gradients that drive the selection of pigmented bacteria with potential roles in environmental degradation.

In this comparative study, three pigmented isolates, namely U2f (from the gills of *Carassius auratus* or a goldfish), THN1 (from the prickles or thorns of the rose plant, *Rosa bourboniana*), and YC (an incidental laboratory isolate), were analyzed for their dye degradation abilities. This research explores the degradation of different dye classes, including Congo red, malachite green, and methylene blue. The findings highlight ecologically diverse, stress-driven reservoirs of pigmented bacteria with potential applications in bioremediation.

## 2 Materials and methods

### 2.1 Sample collection

Samples were collected in triplicate from diverse ecological niches to investigate the diversity of pigmented microflora. A dead goldfish was collected from a private aquarium in Pune, India, with written consent from the owner, and rose prickles were collected from a mature rose plant in a home garden. These samples were collected separately in sterile autoclavable bags and immediately transferred to the laboratory for further processing. The U2f isolate was obtained from the gills of the goldfish (*Carassius auratus*), specifically targeting the mucosal layer of the gill region using sterile forceps. (Disclamier: The current study was conducted solely on a dead goldfish collected from a private aquarium, and the fish was safely burried in soil after completion of experiment). The THN1 isolate was recovered from the rose prickles/thorns (*Rosa bourboniana*). During the isolation process on nutrient agar, a distinct yellow-pigmented bacterial colony was observed as a contaminant and was subsequently isolated as YC for further characterization (Singh et al., 2022).

### 2.2 Isolation and purification of microorganisms

The U2f isolate was recovered from the gills of healthy goldfish (*Carassius auratus*), while the THN1 isolate was recovered as an endophyte from healthy rose prickles/thorns (*Rosa bourboniana*) following surface disinfection with Tween 20, 70% ethanol for 1 min, and sodium hypochlorite for 4 min, as described by Costa et al. (2012). All surface sterilization procedures and isolation experiments were performed in triplicate. Bacteria were isolated from excised gills as well as from rose prickles by using two methods: direct plating of blotted tissue sections and a crude extract method involving serial dilutions (10^−1^ to 10^−9^) in sterile saline. Aliquots (100 μL) were spread onto nutrient agar (NA) using the spread-plate technique and incubated at 30 ± 2 °C for 24–48 h. Morphologically distinct colonies were purified by quadrant streaking and subsequently transferred to King’s B medium to enhance pigment production (Karthiga Rani et al., 2016). During this isolation process, a distinct yellow-pigmented colony was observed as a spontaneous contaminant on the NA plate and was designated as isolate YC. YC (yellow) was purified to homogeneity using the four-quadrant streaking method on a fresh NA plate. The plates were incubated at 30 ± 2 °C for 24–48 h, and pure cultures were maintained at 4 °C for subsequent analysis (Costa et al., 2012; Karthiga Rani et al., 2016).

### 2.3 Morphological and cellular characterization

The isolated strains (U2f, THN1, and YC) were characterized based on their macromorphological and micromorphological features. Colony morphology, including shape, margin, surface texture, and opacity, was observed following incubation on nutrient agar. Special attention was paid to pigmentation, identifying U2f as tan-colored, THN1 as bright orange-colored, and YC as a yellow-pigmented isolate. To examine cell morphology and Gram characteristics, Gram staining was performed to differentiate the isolates based on their Gram reaction (positive or negative), cell shape (cocci or bacilli), and motility (Wanger et al., 2017).

### 2.4 Molecular identification and phylogenetic analysis

To confirm the taxonomic identity of the isolates (U2f, THN1, and YC), molecular characterization was performed using 16S rRNA gene sequencing. Genomic DNA was extracted from pure cultures using the phenol-chloroform method. The 16S rRNA gene was amplified via polymerase chain reaction (PCR) using the universal primers, 27F and 1492R. The PCR thermal cycling conditions consisted of an initial denaturation at 95 °C for 5 min, followed by 30 cycles of denaturation at 95 °C (30 s), annealing at 55 °C (30 s), and extension at 72 °C (90 s), with a final extension at 72 °C for 10 min. The taxonomic identity of the isolates (U2f, THN1, and YC) was confirmed through 16S rRNA gene sequence analysis. The raw sequence data were first trimmed to remove low-quality bases and primer sequences using BioEdit or ChromasPro. The refined sequences were then subjected to a homology search using the Basic Local Alignment Search Tool (BLASTn) against the National Centre for Biotechnology Information (NCBI) GenBank database. Isolates were identified based on the percentage of sequence similarity with validly published strains.

To determine the evolutionary relationships among the isolates, their nucleotide sequences were aligned with closely related reference sequences using the ClustalW alignment tool. Phylogenetic analysis was performed by constructing a tree based on the maximum likelihood method using MEGA software (Version X). The statistical confidence of the phylogenetic tree was assessed by conducting a bootstrap analysis with 1,000 iterations, which provided support for the stability of the observed clustering patterns. The finalized sequences were submitted to the NCBI GenBank database, and corresponding accession numbers were assigned (Tamura et al., 2013).

### 2.5 Pigment extraction and characterization

The extraction of pigments from isolates U2f, THN1, and YC was performed following the modified solvent extraction protocol described by Sasidharan et al. (2010) and Chaturwedi et al. (2024). The isolates were inoculated in Luria-Bertani (LB) broth and incubated under shaking conditions at 30 °C for 72 h at 120 rpm to achieve optimal biomass and pigment concentration. Following the incubation period, the cultures were harvested by centrifugation at 8,000 rpm for 10 min at 4 °C. The resulting supernatant was discarded, and the cell pellet was washed twice with sterile distilled water to remove residual medium. To recover the intracellular pigments, the pellet was resuspended in 95% ethanol and subjected to a water bath treatment at 60 °C for 15 min. This thermal-solvent treatment facilitated the release of pigments from the cellular matrix. The mixture was subsequently centrifuged, and the pigmented supernatant was collected and filtered. The crude extracts were stored in amber vials at 4 °C to prevent photodegradation and were utilized for further characterization via FTIR and bioremediation assays (Sasidharan et al., 2010; Chaturwedi et al., 2024).

### 2.6 Characterization of extracted pigments

To identify the functional groups, present in the pigments, FTIR analysis was performed. The extracted pigment samples were dried into a powder and scanned in the mid-infrared region from 4,000 to 400 cm^−1^ using an FTIR spectrometer. The resulting spectra were analyzed for characteristic vibration bands, such as O-H stretching, C=O carbonyl groups, and aromatic ring vibrations, to characterize the chemical identities of the tan, orange, and yellow pigments (Ahmad et al., 2012).

### 2.7 Screening of the dyes

To evaluate the decolorization and biodegradation potential of the three bacterial isolates, screening was performed against a chemically diverse array of synthetic dyes, including Congo red, malachite green, bromophenol blue, methylene blue, and crystal violet. These dyes represent different structural classes and chromogenic groups. Congo red is an azo dye characterized by –N=N– linkages connecting two aromatic rings. Malachite green and crystal violet are triphenylmethane dyes containing a central carbon atom bonded to three aromatic rings, while methylene blue is a thiazine dye with a heterocyclic aromatic structure containing sulfur and nitrogen atoms, with conjugated double bonds. The decolorization potential was assessed across a broad concentration gradient ranging from 1 mg/mL to 500 mg/mL. This range was selected to determine both the minimum threshold for visible degradation and the maximum tolerance limits (MIC) of the isolates against high dye loading, simulating industrial effluent conditions (Zabłocka-Godlewska et al., 2018).

### 2.8 Dye decolorization and bioremediation potential assessement of the isolates

The bioremediation efficiency of the isolates (U2f, THN1, and YC) was evaluated through a decolorization assay using the synthetic azo dye Congo red and the triarylmethane dye malachite green. The experimental procedure was adapted and modified from the methodology described by Hanis et al. (2020). To assess the tolerance and decolorization capacity of the isolates, a concentration gradient of the respective dyes (1 mg/mL to 500 mg/mL) were established in Nutrient Broth (NB). All experiments were conducted in triplicate, and dye decolorization was confirmed by repeating the experiments on separate days. Moreover, the dye-decolorization potential of the isolates was tested by inoculating individual isolates in nutrient broth, as well as by inoculating consortia in the nutrient medium containing the respective dye concentrations. For example, for Congo red degradation, U2f was inoculated into nutrient broth contained with dye concentrations ranging from 1 mg/mL to 500 mg/mL. For malachite green degradation, THN1 and YC were inoculated individually and as a consortium into medium containing dye concentrations ranging from 1 mg/mL to 500 mg/mL. Consortium preparation briefly included the following steps: for the defined consortium of THN1 and YC, both the cultures were grown separately in nutrient broth to mid-log phase, washed, normalized to a fixed OD or cell count, and combined in equal volumes to prevent any fast-growing strain from dominating immediately. In broth culture, the inoculum density of each isolate was 1% (v/v), with a 1:1 inoculum ratio and an intial optical density of 1.0 (10^6^ cells/mL) before the cultures were pooled in equal proportions. All cultures were incubated at 30 °C, the optimal temperature for growth, at a shaking speed of 90 rpm for 2 days. This aerobic condition was maintained to facilitate efficient biological treatment.

Following the incubation period, the samples were centrifuged at 4,000 rpm for 30 min to remove the biomass. The residual dye concentration in the supernatant was measured using a UV–Vis spectrophotometer (UV-1800 Shimadzu, Japan). According to the standard analytical wavelengths established by Rani and Singh (2021), the maximum absorbance (ʎ_max_) was recorded at 497 nm for Congo red and 617 nm for malachite green. The decolorization efficiency was determined by measuring the absorption intensity at these specific wavelengths, where a decrease in absorbance indicates successful decolorization. Decolorization was monitored by UV–Vis spectroscopic analysis, whereas biodegradation was monitored using FTIR spectroscopy and HPLC (Shimadzu, Japan) (Hanis et al., 2020; Rani and Singh, 2021).

For biodegradation assessment by FTIR, centrifuged samples were dried to obtain an amorphous powder. The powdered samples were then subjected to FTIR analysis to assess dye degradation by the respective isolates against a standard dye concentration of 200 mg/mL or an uninoculated control (Ayed et al., 2011). For HPLC analysis, centrifuged samples after incubation were processed, dissolved in suitable HPLC-grade solvents, and analyzed (Selva Raj et al., 2012; Parshetti et al., 2006).

For Congo red dye degradation analysis by HPLC, chromatographic separations were performed using a Shimadzu prominence i LC 2030 Plus HPLC system equipped with a low-pressure LC-2030 pump, LC-2030 autosampler, and LC-2030 UV detector. Analysis was carried out on a reverse-phase C18 stationary-phase column (250 mm × 4.6 mm, 5 μm particle size). The column temperature was maintained at 30 °C (or at *ambient/*25 °C, as an alternative) to ensure reproducibility. The mobile phase consisted of two components: mobile phase A (aqueous buffer): 35% Milli-Q water (pH adjusted to 5.0–5.8), and mobile phase B (organic modifier), comprising 65% HPLC-grade acetonitrile. A low-pressure gradient program was executed at a constant flow rate of 2.0 mL/min, with an injection volume: 10–20 μL, over a total run time of 20 min; absorbance was recorded at 680 nm (Selva Raj et al., 2012). A primary stock solution of pure analytical-grade Congo red (purity >95%) was prepared in HPLC-grade water/methanol. Working standard calibration solutions were generated across a linear range of 1.0–100.0 mg/L via serial dilution. Calibration curves were constructed by plotting the integrated peak areas against nominal concentrations, showing high linearity with a correlation coefficient (R^2^ > 0.999). The detection-limit coefficient was 3.3, and the quantitation-limit coefficient was 10.0. Chromatographic peaks were automatically integrated using the Chromatopac algorithm. For metabolite identification, chromatographic profiles were analyzed based on degraded compounds and their retention times after setting the required wavelength and time.

For malachite green dye degradation analysis by HPLC, the protocol specification was as follows: with a C18 reverse-phase column (125 mm × 4.60 mm) was used, with a gradient consisting of mobile phase A (60% Milli-Q water) and mobile phase B (40% methanol) at a flow rate of 0.5 mL/min, followed by 10-min linear gradient to 95% ethanol. This gradient was held for an additional 8 min at the same flow rate, with an injection volume of 15 μL, and absorbance was recorded at 620 nm (Parshetti et al., 2006). The remaining specifications were kept the same as those used for the Congo red HPLC analysis.

### 2.9 Statistical analysis

All experiments were conducted in triplicate, and the results were expressed as mean ± standard deviation (SD). The data were graphically represented using Microsoft Excel. Line graphs were plotted using the mean values, with error bars representing the standard deviation, to facilitate clear visualization and comparison of treatment effects across different experimental conditions.

## 3 Results

### 3.1 Isolation and morphological characterization of pigmented bacteria

Three distinct pigmented bacterial strains were isolated from diverse ecological niches. The isolate U2f, obtained from goldfish gills, showed tan-colored colony morphology. Isolate THN1, isolated from rose prickles/thorns, showed bright orange-colored colony morphology, while isolate YC, an incidental laboratory isolate, showed a characteristic yellow-colored colony morphology. The colony characteristics on nutrient agar (NA) and the microscopic results of Gram staining are summarized in Table 1. The visual representation of the colony morphologies is provided in Figure 1.

**Table 1:** Morphological characterization of pigmented isolates.

Isolates name	U2f	YC	THN1
Source	Goldfish gills	Incidental laboratory isolate	Rose prickle/thorn
Colony size	1 mm	2 mm	2 mm
Colony shape	Circular	Circular	Circular
Color	Tan	Yellow	Bright orange
Surface	Smooth	Smooth	Smooth
Elevation	Slightly convex	Convex	Convex
Opacity	Translucent	Opaque	Opaque
Margin	Entire	Entire	Entire
Gram nature	Gram negative coccobacilli	Gram positive cocci	Gram negative cocci in pairs
Motility	Actively motile	Non-motile	Non-motile
Catalase test	Positive	Positive	Positive
Oxidase test	Positive	Positive	Positive

**Figure 1:**
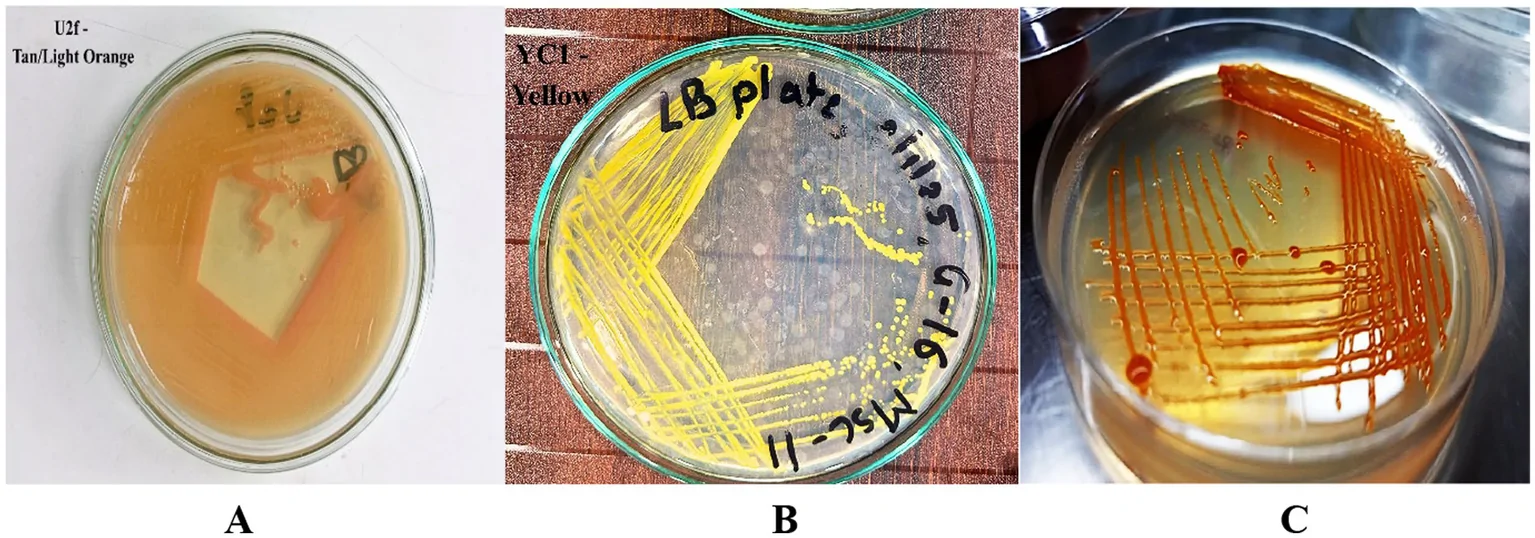
Colony morphology of pigmented bacterial isolates on nutrient agar media. Isolate U2f exhibited tan to light orange pigmented colonies **(A)**. In contrast, isolate YC displayed characteristic bright yellow colonies **(B)**, and isolate THN1 produced bright orange colonies **(C)**.

### 3.2 Molecular identification and phylogenetic analysis

To confirm the taxonomic identity of the isolates, molecular characterization was performed using 16S rRNA gene sequencing. Genomic DNA was extracted, and the 16S rRNA gene was successfully amplified by PCR using universal primers 27F and 1492R. The presence of a distinct DNA fragment at the expected size of approximately 1,500 bp, confirmed amplification of the 16S rRNA gene in all three isolates. The purified amplicons were subsequently subjected to Sanger sequencing to determine their taxonomic identity.

The consensus sequences obtained from Sanger sequencing were analyzed using the BLASTn program against the NCBI database. Based on a sequence similarity of ≥99%, the isolates were identified as follows: U2f was identified as *Shewanella xiamenensis*, the incidental laboratory isolate YC was identified as *Kocuria* sp., and THN1 was identified as *Paracoccus* sp. For the isolate THN1, the sequence similarity was only 96.28%. According to the authors, sequence similarity values between 95 and 98.65% generally indicate that a sequence belongs to the correct genus but may represent a completely new, undocumented species. Therefore, the prickle/thorn endophyte THN1 may represent a novel species within the genus *Paracoccus.* However, detailed genomic identification is neccessary to establish whether THN1 represents a new species from this unconventional niche. The molecular identification for all isolates, including percentage similarity and query coverage, is summarized in Table 2. The phylogenetic trees constructed of all isolates are shown in Figures 2A–C.

**Table 2:** Molecular identification of pigmented isolates.

Isolates	Closest taxonomic relative	% Similarity	Query coverage	GenBank accession
U2f	*Shewanella xiamenensis*	99.59%	100%	PZ276692
YC	*Kocuria* sp.	99.93%	100%	PX778014
THN1	*Paracoccus* sp.	96.28%	100%	OQ923795

**Figure 2:**
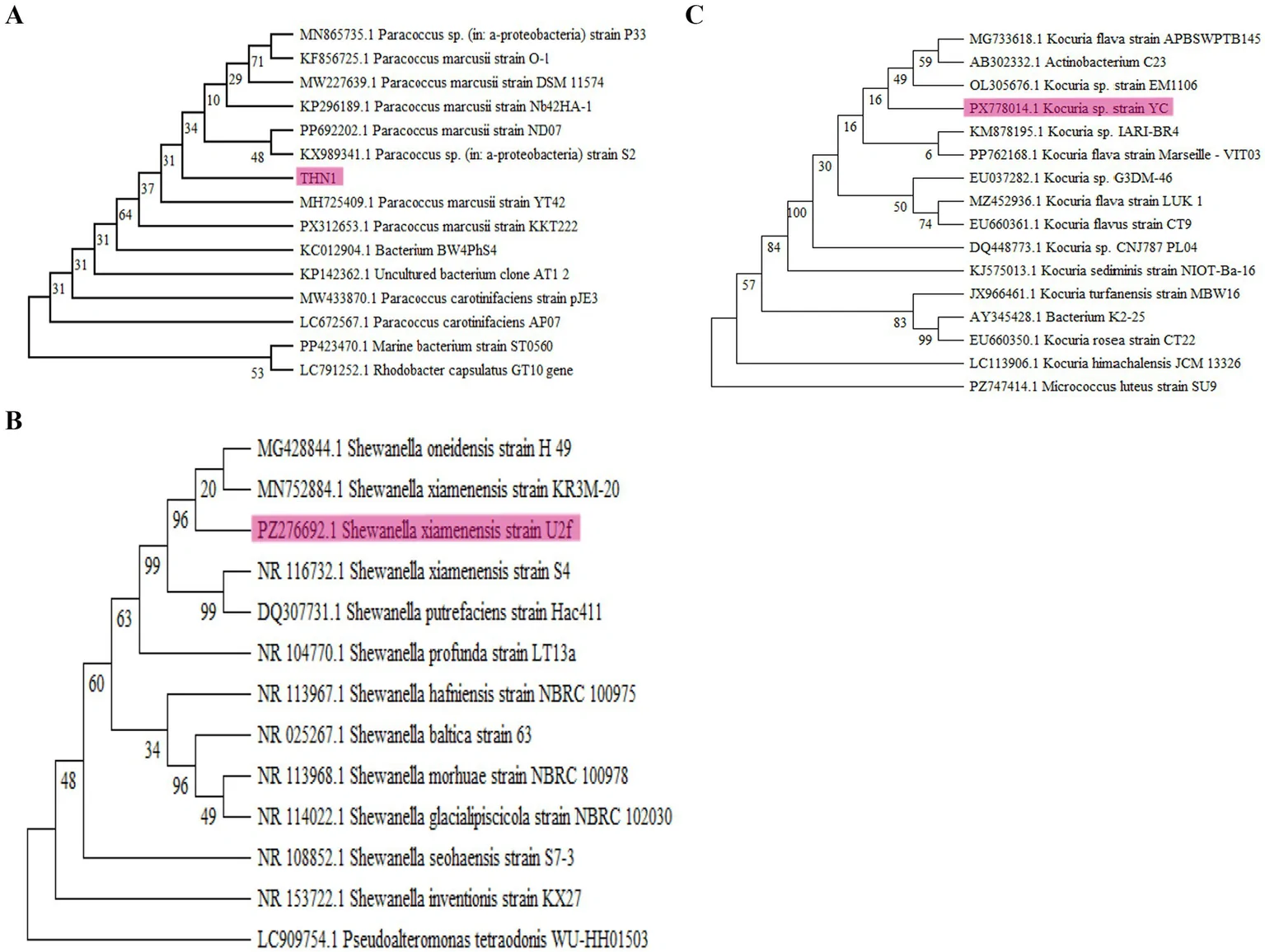
**(A)** Phylogenetic analysis of prickle/thorn endophyte THN1 by maximum likelihood method. **(B)** Phylogenetic analysis of goldfish gill isolate U2F by maximum likelihood method. **(C)** Phylogenetic analysis of an incidental laboratory isolate YC by maximum likelihood method.

### 3.3 Phylogenetic tree for pigmented isolates

### 3.4 Characterization of pigments

FTIR analysis of the extracted pigment revealed characteristic absorption bands corresponding to different functional groups. The observed absorption patterns were consistent with the reported profiles of melanin and β-carotene. Based on the spectral features and comparison with the standards, the pigment obtained from U2f (*Shewanella xiamenensis*) showed similarity to melanin (Figures 3a,b) the pigment from YC (*Kocuria* sp.) showed characteristics consistent of β-carotene (Figures 4a,b), whereas the pigment obtained from THN1 (*Paracoccus* sp.) showed similarity to astaxanthin (Figures 5a,b).

**Figure 3:**
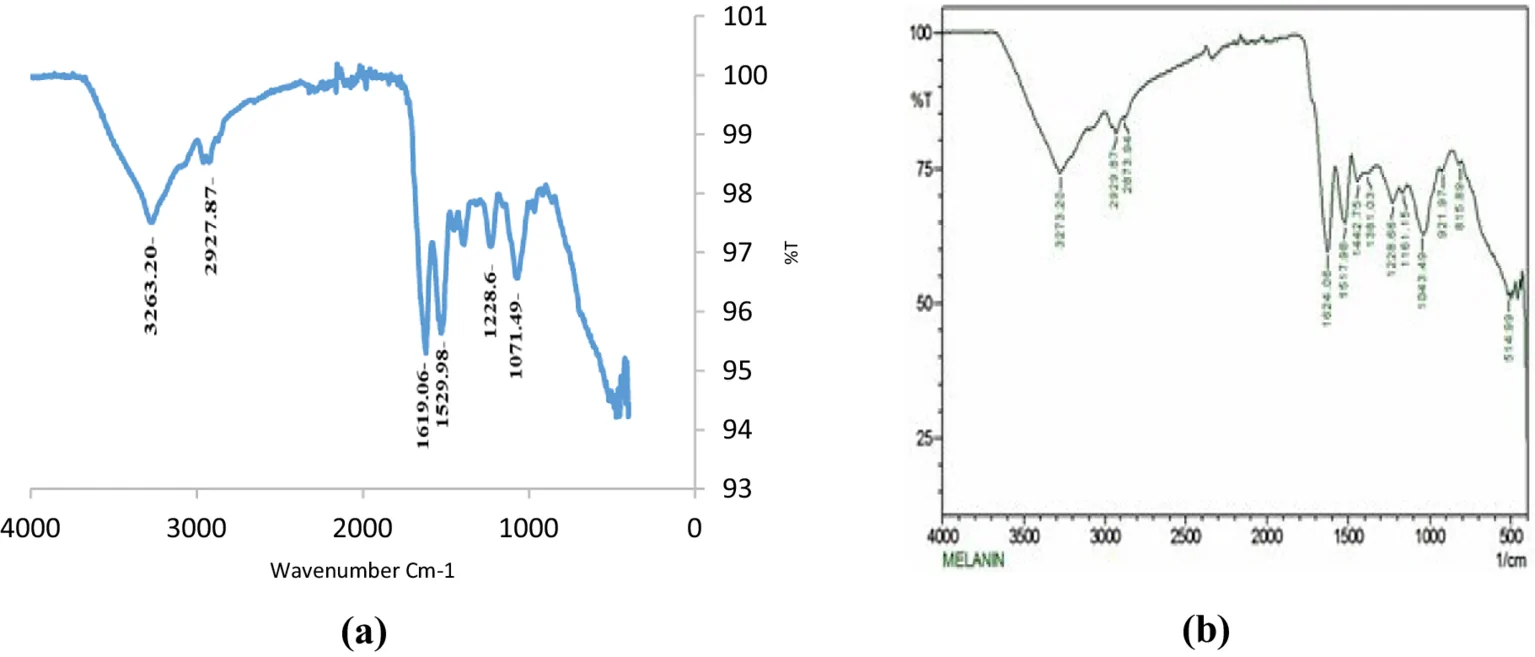
**(a)** FTIR spectra of U2f pigment; **(b)** Standard FTIR spectra of melanin (Kiran et al., 2017).

**Figure 4:**
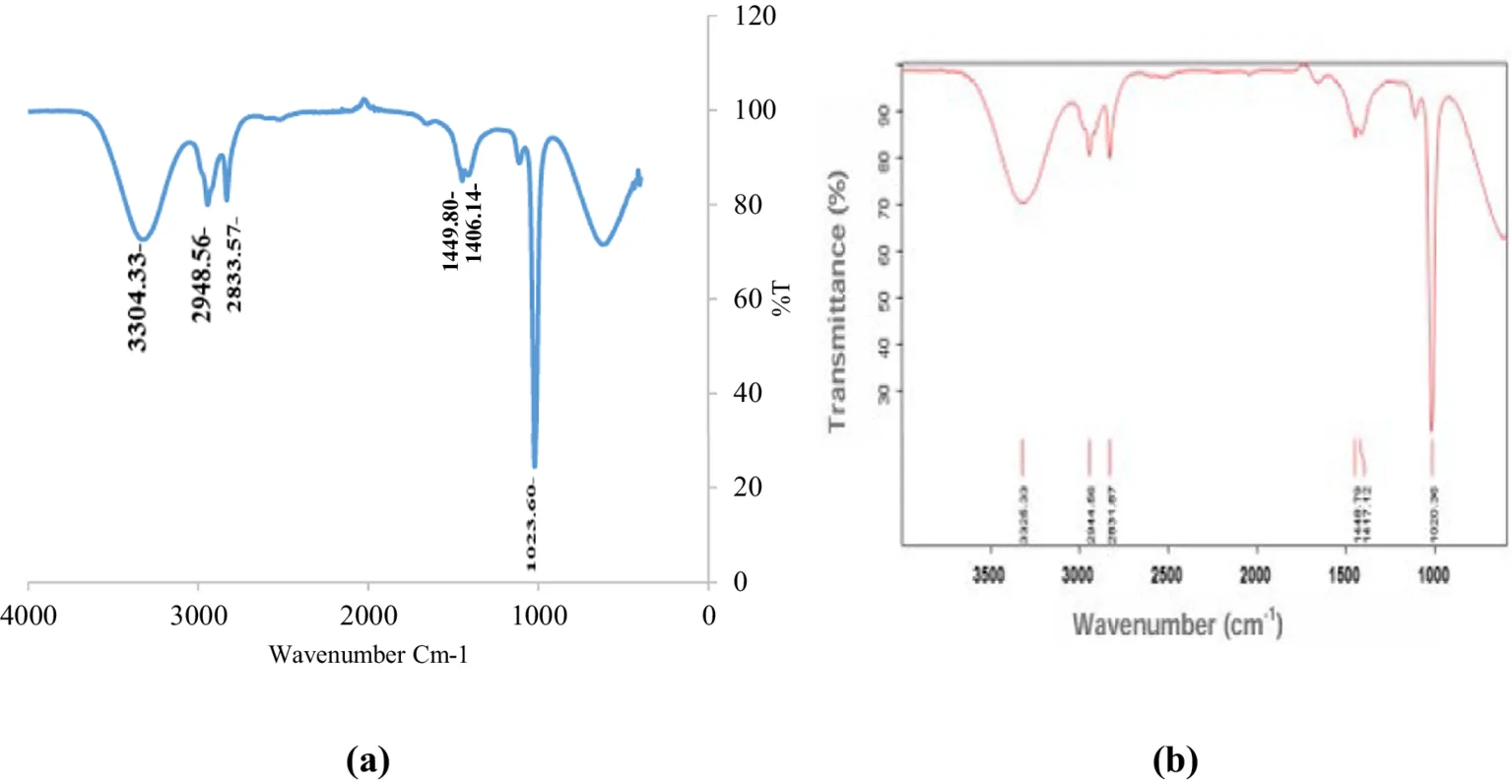
**(a)** FTIR spectra of YC pigment; **(b)** Standard FTIR spectra of beta-carotene pigment (Neha et al., 2017).

**Figure 5:**
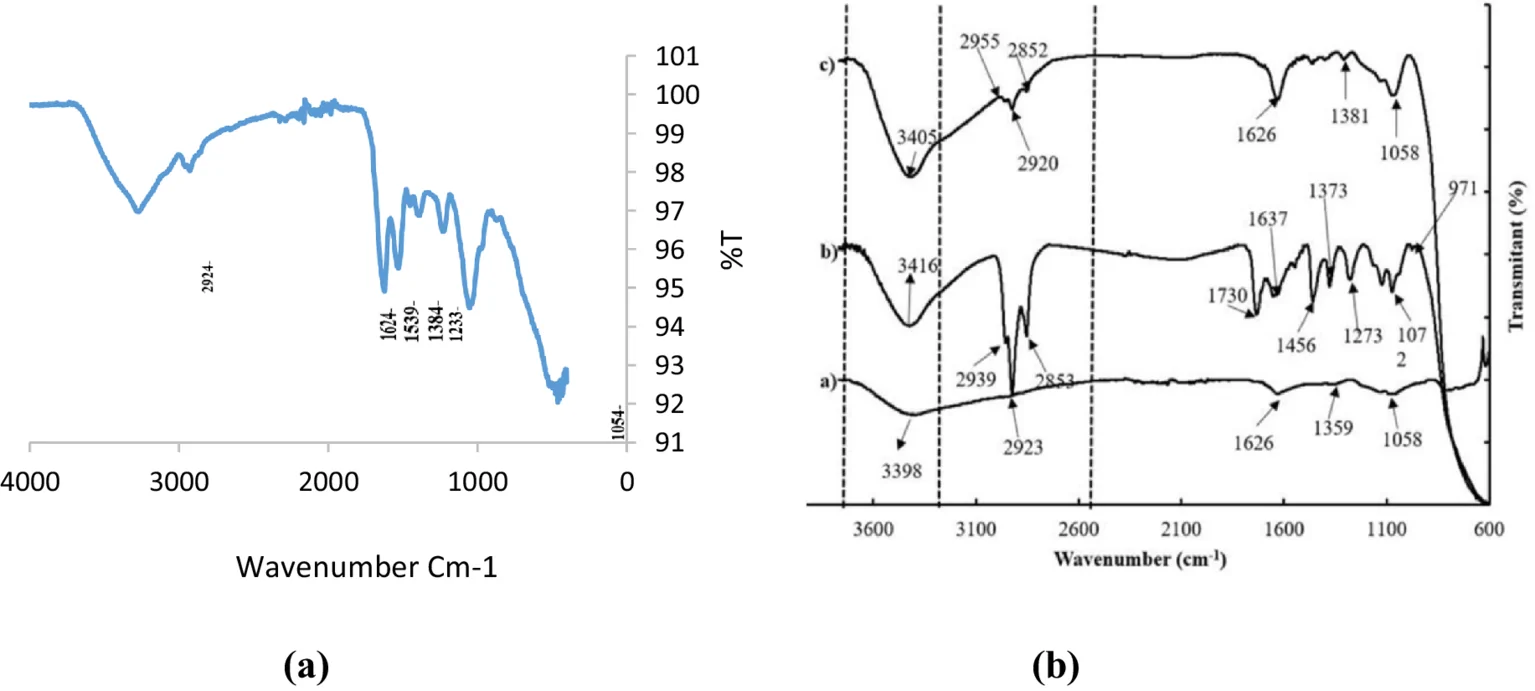
(a) FTIR spectra of THN1 pigment; **(b)** Standard FTIR spectra of astaxanthin pigment (Septiani et al., 2024).

### 3.5 Evaluation of dye specificity and decolorization range

The three bacterial isolates were screened against a diverse selection of synthetic dyes to determine their decolorization range. Although the isolates were tested across a wide concentration gradient of 1 mg/mL to 500 mg/mL, they exhibited highly selective activity. Isolate U2f demonstrated the ability to effectively decolorize Congo red, indicating its potential to degrade the azo-linked dye structure. In contrast, no decolorization of Congo red was observed for the other two isolates, THN1 and YC. Malachite green decolorization was observed for isolates YC and THN1, both of which exhibited the ability to decolorize the triphenylmethane dye. However, isolate U2f did not show any noticeable activity aganist malachite green.

None of the isolates showed significant decolorization for bromophenol blue, methylene blue, or crystal violet, indicating no decolorization activity aganist these dye classes within the experimental concentration range of 1–500 mg/mL. Successful decolorization was observed exclusively for Congo red and malachite green.

The isolates demonstrated significant but distinct bioremediation efficiencies against the two classes of synthetic dyes tested. Decolorization was quantified by measuring the reduction in absorbance at the specific ʎ_max_ for each dye, namely, 497 nm for Congo red and 617 nm for malachite green, as shown in Figure 6. The decolorization efficiency was calculated using the following equation:

$$\text{Decolorization efficiency}\;(\%)=A_{0}–A_{t}/A_{0}\times 100$$

Where A_0_ = absorbance of the incubated abiotic control and A_t_ = absorbance of the test sample.

**Figure 6:**
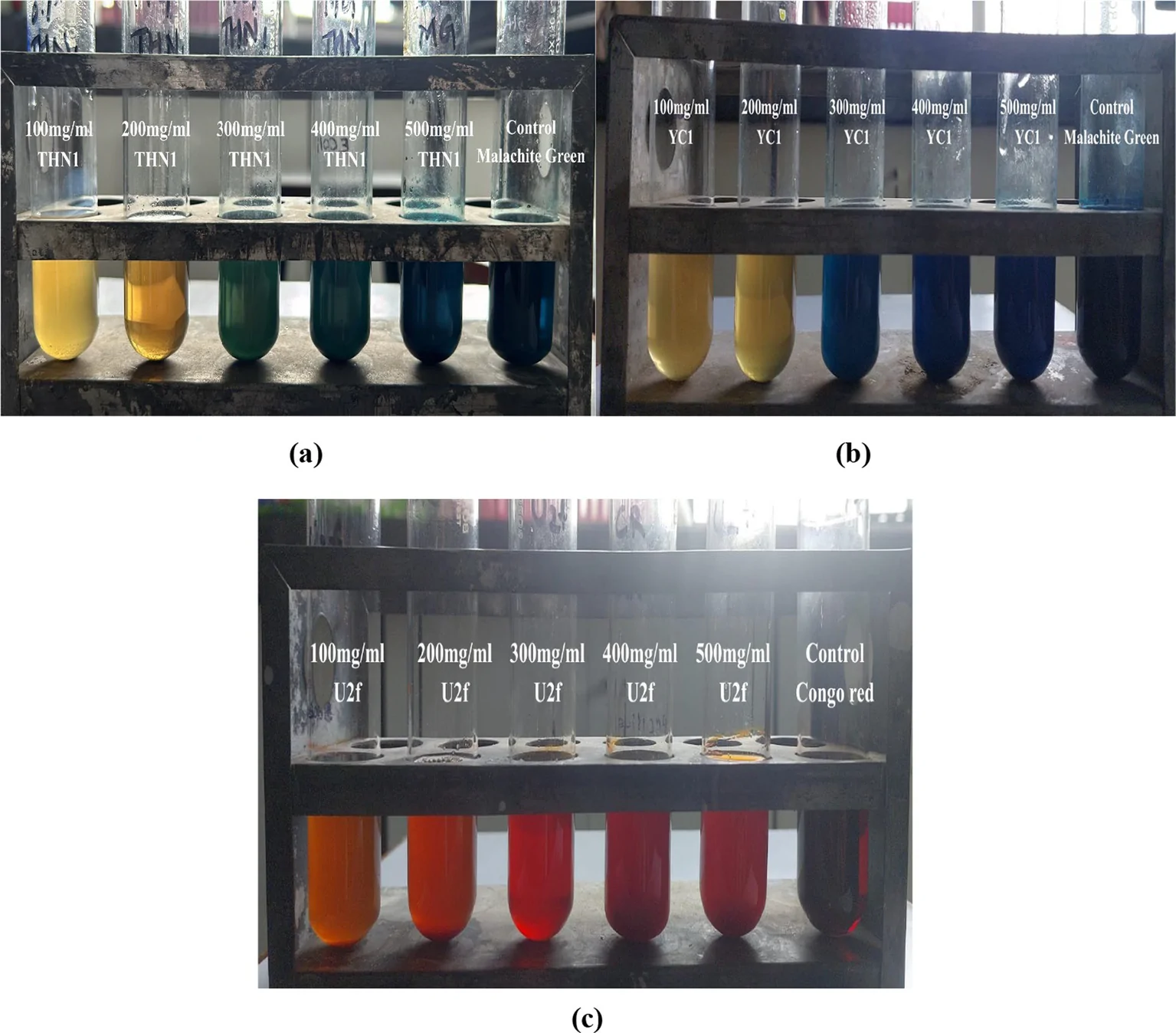
Visual observation of dye decolorization **(a)** Decolorization of malachite green dye by isolate THN1- *Paracoccus* sp., **(b)** Decolorization of malachite green dye by isolate YC – *Kocuria* sp. and **(c)** Decolorization of Congo red by isolate U2f – *Shewanella xiamenensis*.

To eliminate optical interference from bacterial biomass and cell-bound pigments during analysis, all aliquots were subjected to high-speed centrifugation (e.g., 10,000 rpm for 10 min) and/or filtration before spectrophotometric assessment. Consequently, the observed reduction in absorbance was intended to reflect extracellular decolorization rather than masking effects caused by pigmented biomass. Although a specific control for pigmented bacteria without dye was not monitored continuously, the maximum absorbance (λmax) of the targeted synthetic dye (X nm) is distinctly separated from the characteristic absorbance spectrum of the bacterial pigment (Y nm). Therefore, any potential secretion of endogenous biopigments by the strain was considered unlikely to interfere with the spectrophotometric quantification of dye degradation.

#### 3.5.1 Decolorization of an azo dye (Congo red) by U2f

*Shewanella xiamenensis* (U2f) exhibited a robust ability to decolorize Congo red at concentrations ranging from 1 to 200 mg/mL. It maintained efficient dye tolerance across a concentration gradient from 300 to 500 mg/mL. The decrease in absorption intensity at 497 nm suggests that the melanin-producing isolate possesses the necessary mechanisms to disrupt the complex azo (-N=N-) bonds of the dye. *Shewanella xiamenensis* (U2f) effectively decolorized Congo red dye with 70% efficiency at the higher concentration of 200 mg/mL, as shown in Figure 7a. However, at lower Congo red concentration of 1–50 mg/mL, the decolorization efficiency of U2f ranged from 81 to 94%, as depicted in Figures 7b,c.

**Figure 7:**
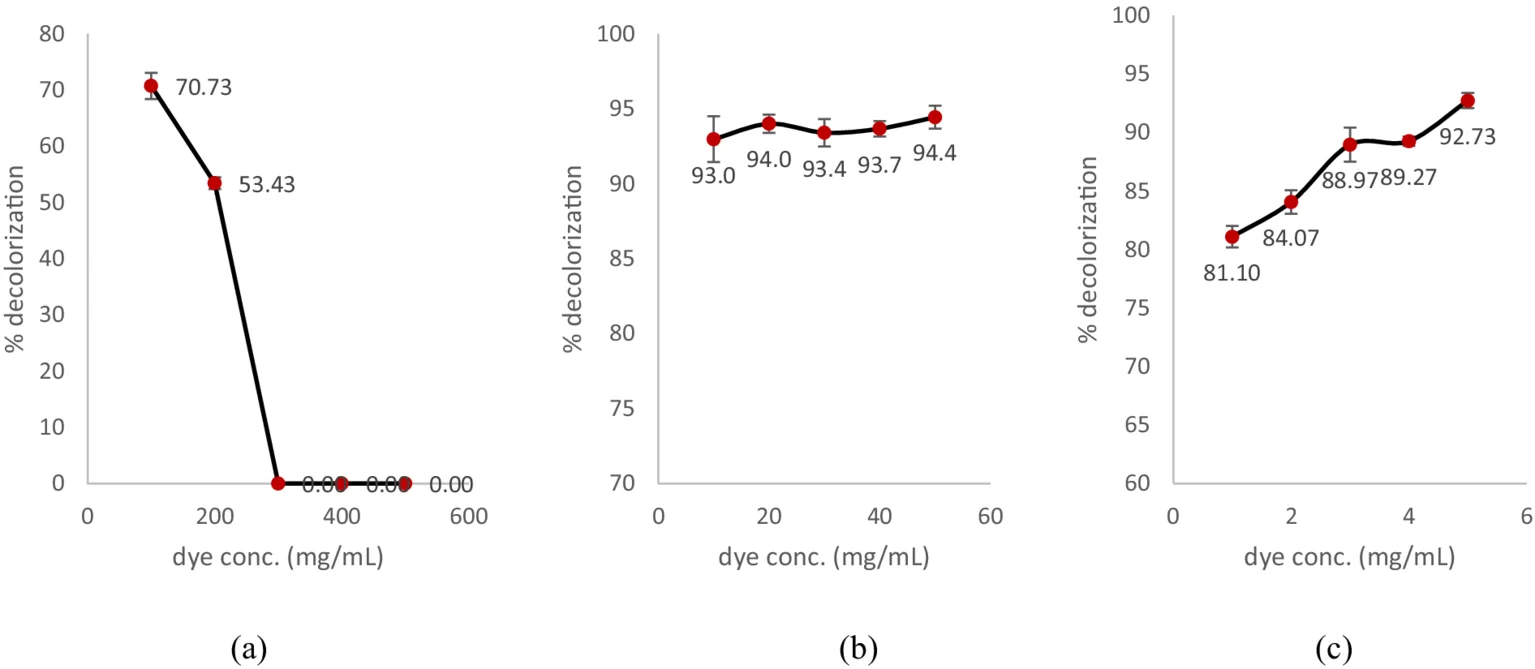
% decolorization of Congo Red dye at concentration ranging from 1 mg/mL to 500 mg/mL: Graph shows percent decolorization of Congo Red dye by U2f at different concentrations, **(a)** for 100–500 mg/mL, **(b)** for 10–50 mg/mL, and **(c)** for 1–5 mg/mL.

#### 3.5.2 Decolorization of triphenylmethane dye (malachite green)

Isolates *Paracoccus* sp. (THN1) and *Kocuria* sp. (YC) were found to be highly effective in the decolorization of malachite green over a wide concentrationa range of 1–500 mg/mL. Both strains showed high tolerance at concentrations ranging from 300 to 500 mg/mL. In contrast to *Shewanella xiamenensis* (U2f), these isolates showed specificity toward the triarylmethane structure of malachite green.

*Paracoccus* sp. (THN1) effectively decolorized malachite green with approximately 91% efficiency, even at a high dye concentration of 200 mg/mL, as shown in Figure 8a. Moreover, *Paracoccus* sp. (THN1) was also found to tolerate the highest dye concentration, i.e., 500 mg/mL with 9.63% decolorization efficiency. In contrast, at lower dye concentration of 1–50 mg/mL, the decolorization efficiency was approximately 57–90%, indicating the potential of this organism to grow and reproduce in an environment consisting high concentrations of dye pollutants (Figures 8b,c).

**Figure 8:**
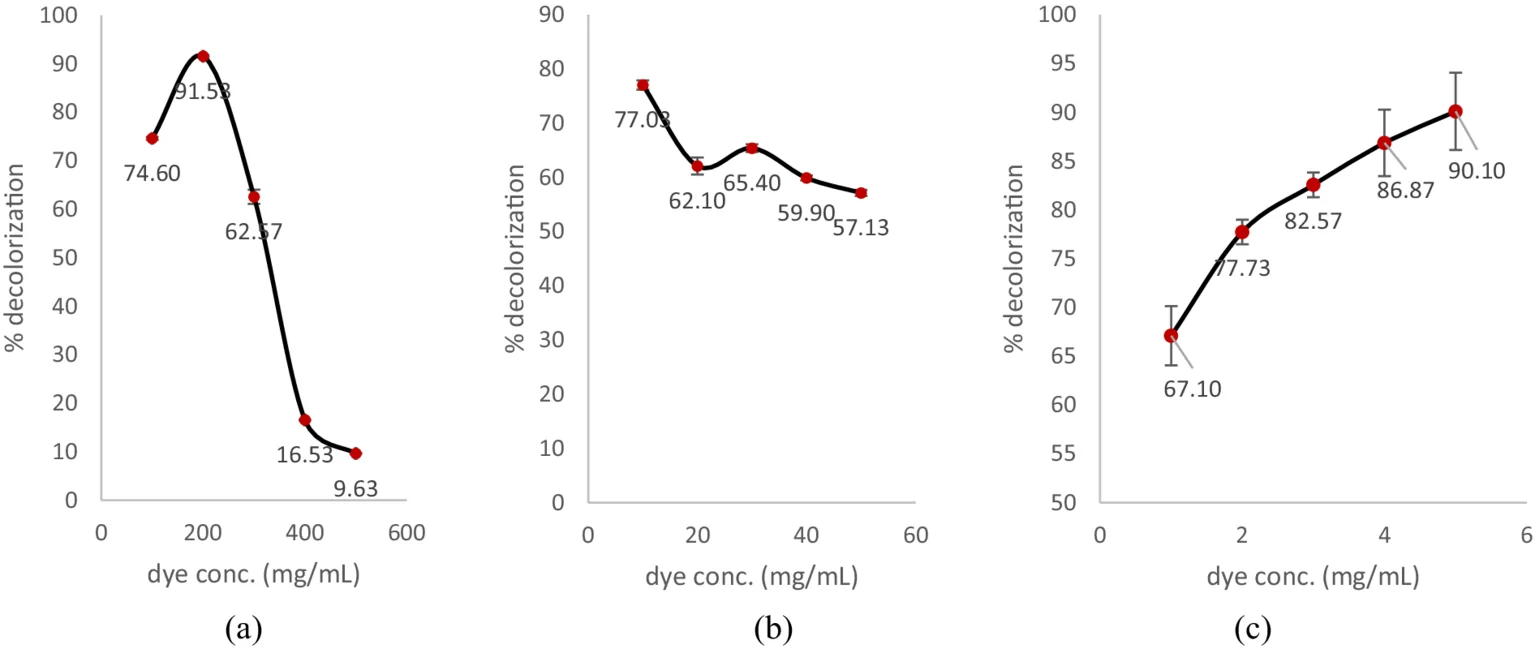
% Decolorization of malachite green dye at concentration ranging from 1 mg/mL to 500 mg/mL by THN1: Graph shows % decolorization of malachite green dye by THN1 at different concentrations, **(a)** for 100–500 mg/mL, **(b)** for 10–50 mg/mL and **(c)** for 1–5 mg/mL.

Similar trends in the malachite green decolorization pattern were observed with *Kocuria* sp. (YC). At higher concentrations of 100–500 mg/mL, the percentage decolorization was as high as 89%, as shown in Figure 9a. At concentrations of 10–50 mg/mL, *Kocuria* sp. (YC) showed a maximum decolorization efficiency of 73% as depicted in Figure 9b. At lower malachite green concentrations, the percentage decolorization was as high as 71%, as depicted in Figure 9c.

**Figure 9:**
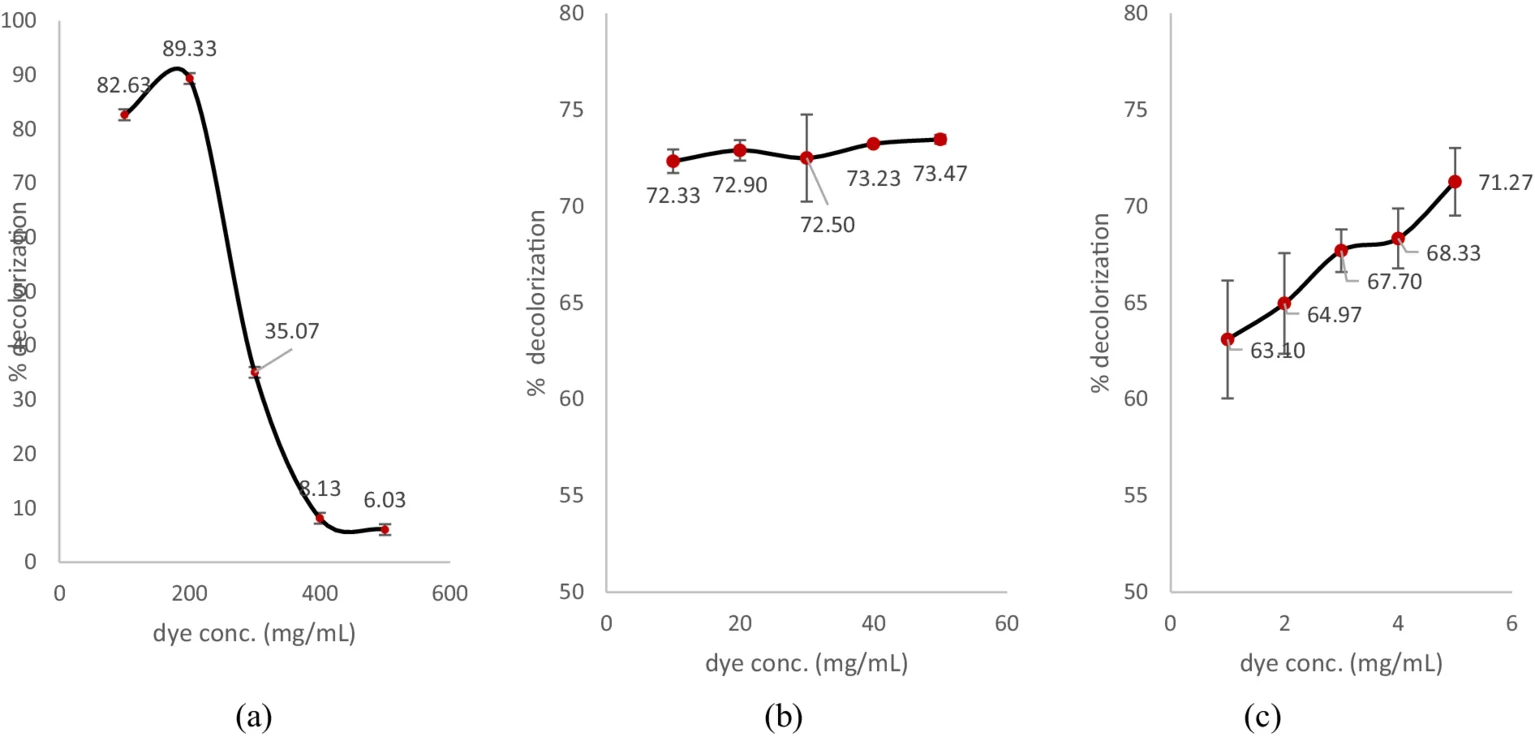
% Decolorization of malachite green dye at concentration ranging from 1 mg/mL to 500 mg/mL by YC: Graph shows % decolorization of malachite green dye by YC at different concentrations, **(a)** for 100–500 mg/mL, **(b)** for 10–50 mg/mL and **(c)** for 1–5 mg/mL.

#### 3.5.3 Dye degradation analysis by using Fourier transform infrared spectroscopy (FTIR)

##### 3.5.3.1 Congo red dye degradation by *Shewanella xiamenensis* (U2f)

Congo red dye degradation by U2f isolate was further confirmed by FTIR analysis of the control and treated groups. The control group contained only dye solution (200 mg/mL), whereas the treated group contained dye solution with the U2f isolate. After 2 days of incubation, the residual degradation products were analyzed using FTIR, which showed peaks presented in Figure 10.

**Figure 10:**
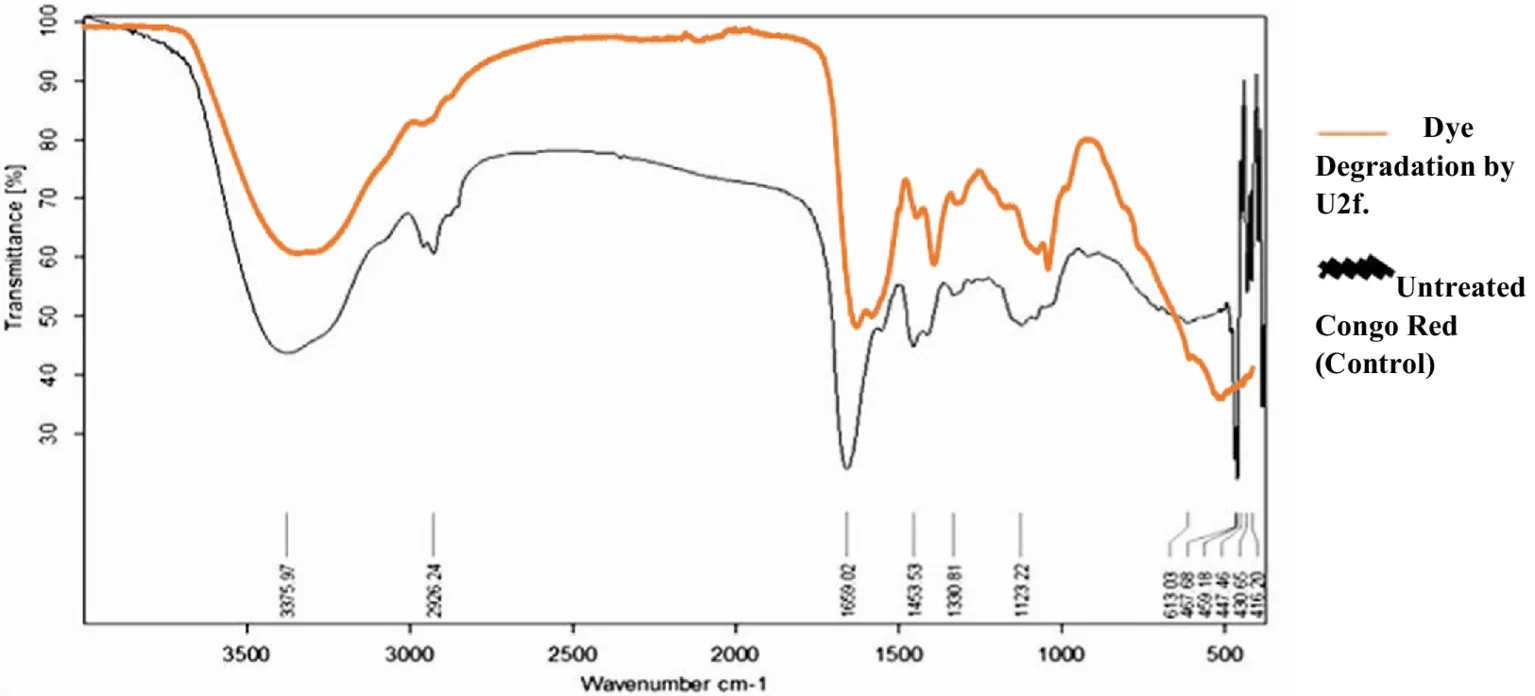
FTIR spectra of control (Congo red) as well as dye degradation by *Shewanella xiamenensis* (U2f).

The signature peaks at 1620 cm^−1^ and 1,598 cm^−1^ were attributed to the presence of azo linkages and N=N stretching, respectively. The distinct peaks from 3,250 cm^−1^ to 2,980 cm^−1^ were attributed to C–H stretching of aromatic compounds. The C ≡ N and C ≡ C stretching bands, along with the peak at 765 cm^−1^, demonstrated the aromatic nature of Congo red. The FTIR spectrum of the dye after treatment with the test organism showed distortion of the signature peaks at 1620 cm^−1^ and 1,598 cm^−1^, indicating cleavage of the azo linkage. In addition, peaks observed in the degraded sample at 1096 cm^−1^ and 1,371 cm^−1^ indicated S–O and S=O stretching, respectively, suggesting degradation of the dye (Chakravarthi et al., 2021).

##### 3.5.3.2 Malachite green dye degradation by *Paracoccus* sp. (THN1) and *Kocuria* sp. (YC)

Similar trends in dye degradation were also observed for malachite green following treatment with *Paracoccus* sp. (THN1) and *Kocuria* sp. (YC). FTIR analysis was performed for the control and treated groups. The control group contained only dye solution (200 mg/mL), whereas the treated groups contained dye solution treated separately with THN1 and YC isolates. After 2 days of incubation, the residual products of degradation were analyzed FTIR technique which showed peaks as shown in Figure 11 (for THN1) and Figure 12 (for YC).

**Figure 11:**
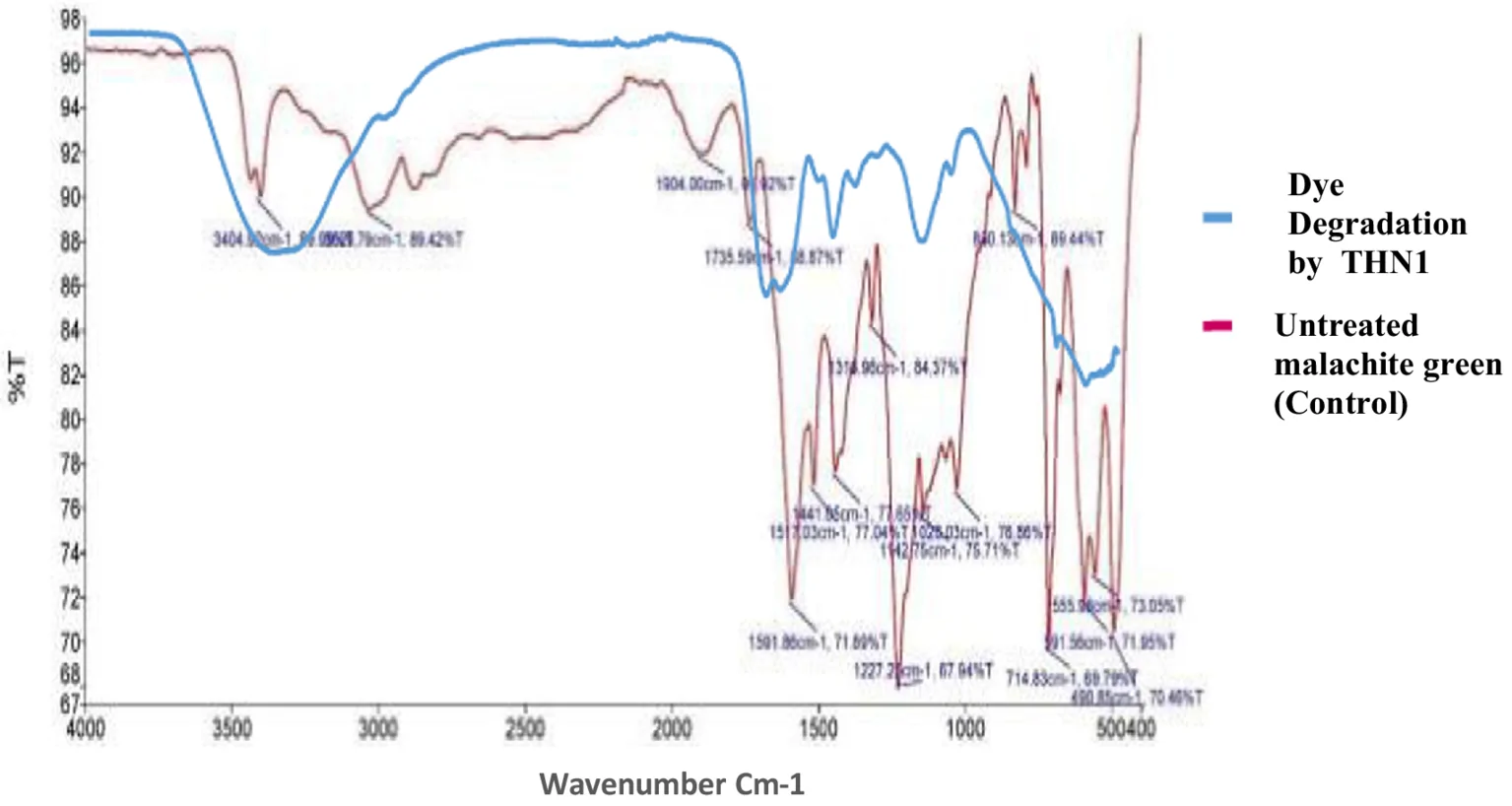
FTIR spectra of control (Malachite green) as well as dye degradation by *Paracoccus* sp. (THN1).

**Figure 12:**
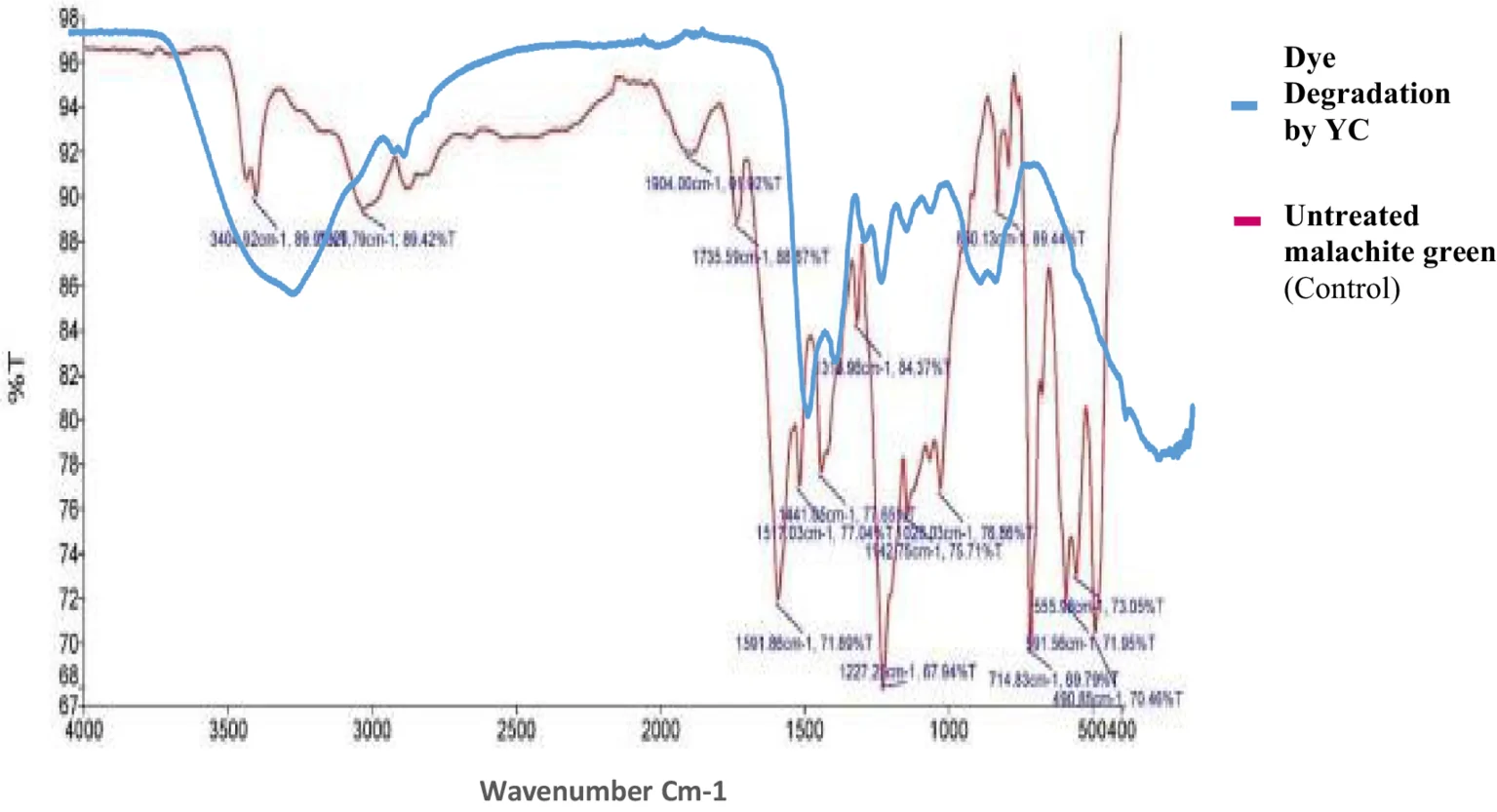
FTIR spectra of control (Malachite green) as well as dye degradation by *Kocuria* sp. (YC).

The FTIR spectrum of the malachite green control showed several peaks in the fingerprint region (1,500–500 cm^−1^). The appearance of some new peaks and the disappearance of peaks important for the structural integrity of the dye were observed in the FTIR spectra samples treated with the isolates. The prominent peak at 1590–1600 cm ^−1^ was reduced in both isolates, indicating a change in the aromatic ring structure of malachite green. The disappearance of the peak at 1318 cm^−1^ indicates that bond between the aromatic ring the nitrogen atom is cleaved. The distortion of the peaks is observed in the fingerprint region as well which confirms the degradation of the malachite green dye by both the organisms *Paracoccus* sp. (*THN1*) and *Kocuria* sp. (YC) (Kaushik and Seth, 2021).

#### 3.5.4 Dye degradation analysis using high performance liquid chromatography (HPLC)

##### 3.5.4.1 High performance liquid chromatography (HPLC) analysis of degradation of Congo red dye

To confirm the degradation of Congo red dye by the isolate *Shewanella xiamenensis* (U2f), HPLC analysis was performed. Whole cells collected from the culture broth after incubation, processed and analysis at absorbance 680 nm was performed for control and test samples. The control chromatogram exhibited two distinct parent peaks at retention time of 2.017 min and 2.575 min. In contrast, in the treated sample the second peak at 2.575 min was significantly diminished, while the primary peak slightly shifts to 2.042 min with a massive increase in peak area, showed prominent peaks at 2.867 min and 3.658 min of completely new metabolites. The slight shift in retention time and altered chromatographic profile indicates the biotransformation of the Congo red dye by U2f. The observed shift in retention time and variation in chromatographic pattern between control and treated samples indicate that bacterial treatment altered the chemical composition of the dye, suggesting possible biotransformation during incubation. The HPLC results (Figure 13) suggests that the *Shewanella xiamenensis* (U2f) have degraded the complex dye during the incubation period, leading to measurable changes in chromatographic behavior.

**Figure 13:**
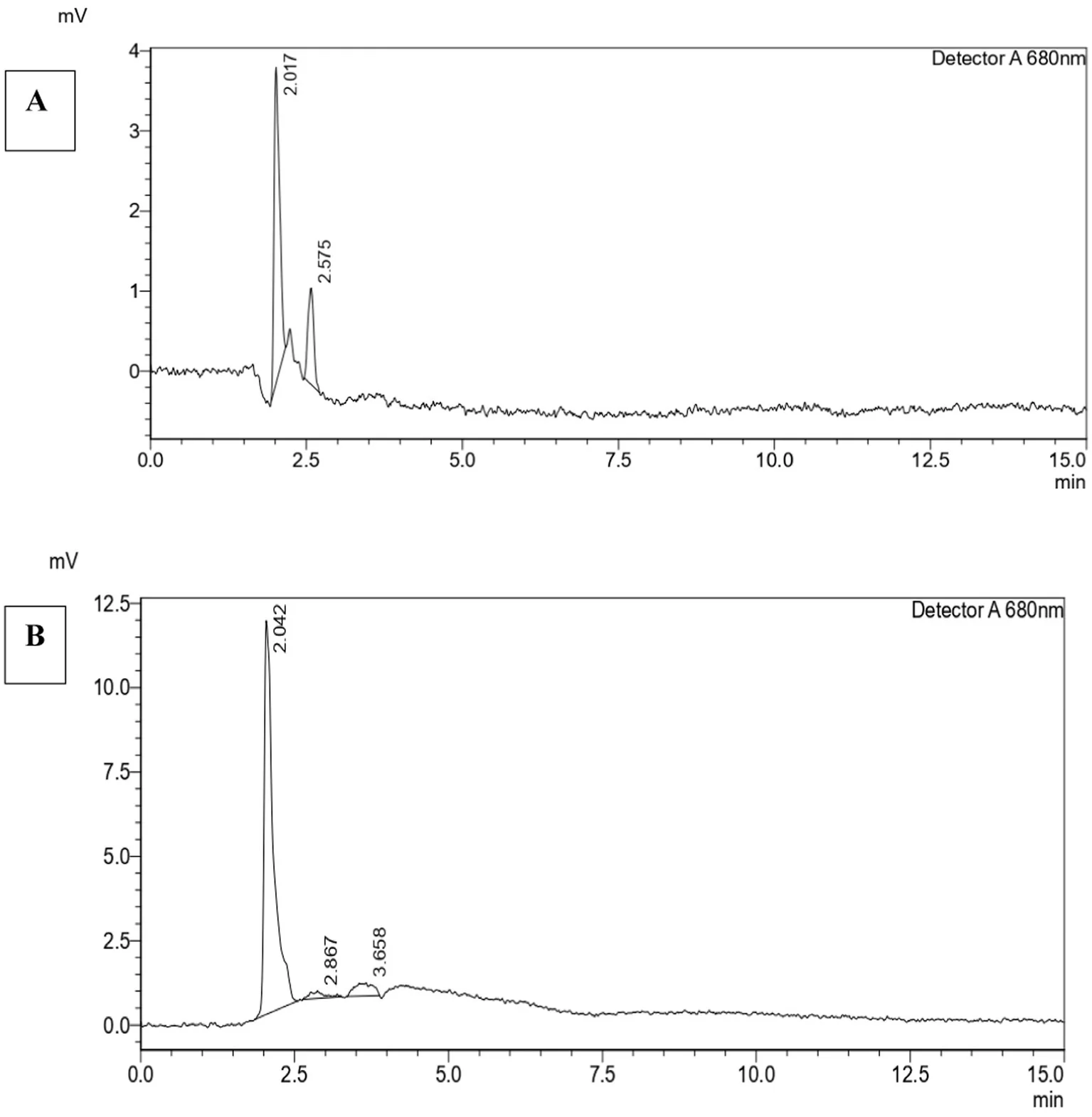
HPLC chromatograms of Congo Red dye degradation. **(A)** Abiotic control (CRC); **(B)** Metabolite profile after *Shewanella xiamenensis* (U2f) treatment showing new peaks at 2.867 and 3.658 min.

##### 3.5.4.2 High performance liquid chromatography (HPLC) analysis of degradation of malachite green dye

The biotransformation of the triphenylmethane dye malachite green using a bacterial consortium of *Paracoccus* sp. (THN1) and *Kocuria* sp. (YC) was confirmed by HPLC analysis. The same procedure used for Congo red was followed, and analysis was performed at an absorbance wavelength of 616–620 nm for the control and test samples. The control chromatogram of malachite green was characterized by prominent sharp peaks at a retention time of 4.925 min and 5.933 min. The major parent peak at 4.925 min was completely eliminated indicating a breakdown of the compound. Concurrently, three distinct intermediate peaks were observed at a retention time of 4.750 min, 5.175 min and 5.500 min. The secondary peak at a retention time of 5.933 min persisted in the treated sample results, but its peak area was slightly reduced from 2,339 to 2035. The observed shift in retention time, absolute disappearance of the peaks and the generation of a cluster of new metabolite peaks in chromatographic pattern (Figure 14) between control and treated samples, chemically confirms that the *Paracoccus* sp. (THN1) and *Kocuria* sp. (YC) consortium actively degraded malachite green during incubation period.

**Figure 14:**
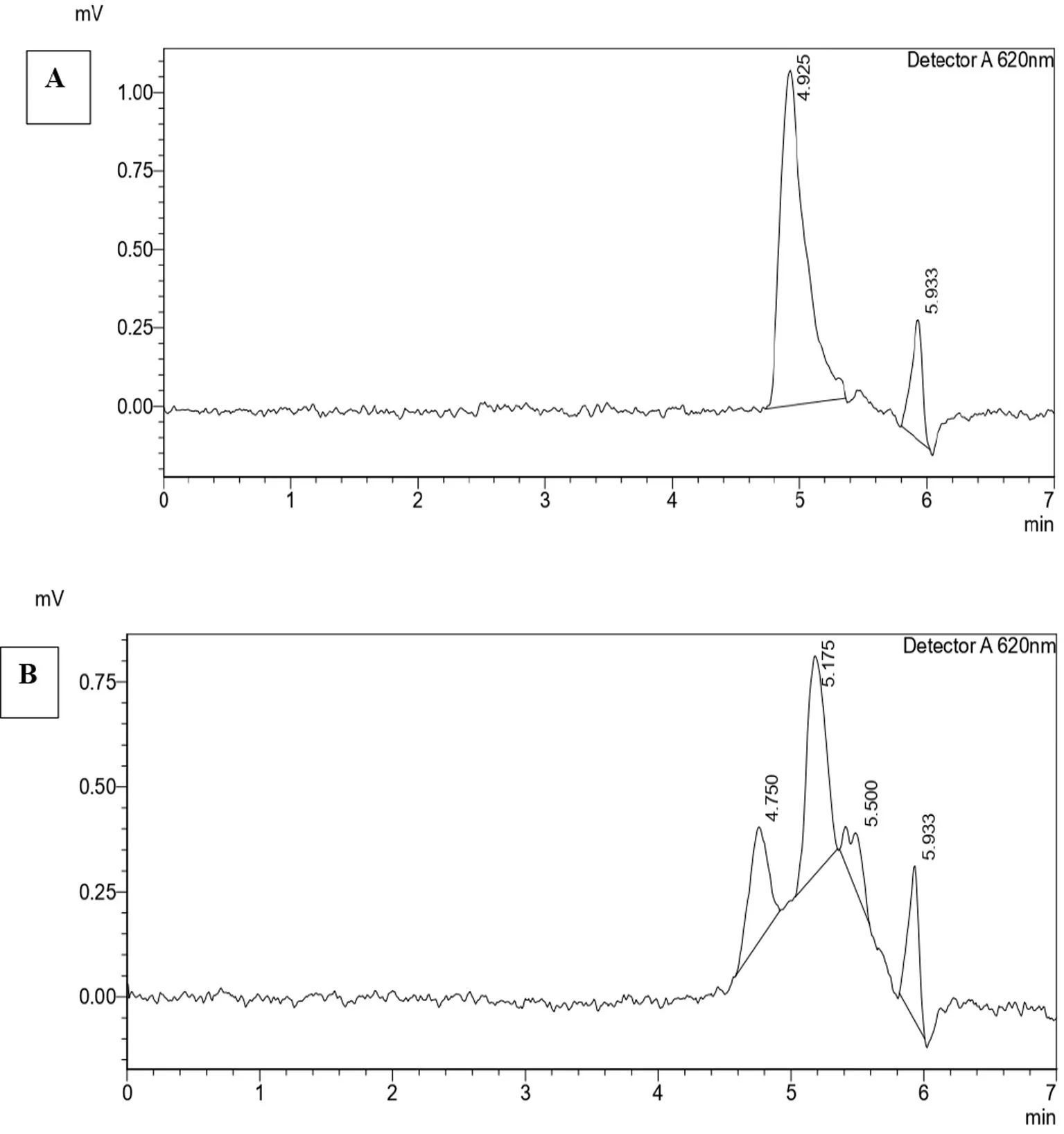
HPLC chromatograms of malachite green dye degradation. **(A)** Abiotic control (MGC); **(B)** Metabolite profile after *Paracoccus* sp. – *Kocuria* sp. (THN1-YC) consortium treatment showing new peaks at 4.750, 5.175, and 5.500 min.

While individual monoculture kinetics were not quantified, the efficacy of the integrated consortium was validated through comprehensive metabolite analysis by HPLC. The complete disappearance of the parent dye coupled with the reduction in total organic carbon (TOC) confirms that the consortium successfully achieved deep mineralization—a feat typically unachievable by isolated monocultures due to the accumulation of toxic dead-end metabolites.

## 4 Discussion

The aim of this study was to isolate and characterize pigmented bacteria with dye-degradation potential from varied ecological niches. As a result, two different pigmented bacteria were successfully isolated from different niches like goldfish gills and rose prickles/thorns. While performing this study one pigmented bacteria was accidently reported on nutrient medium which was also later included in the current study. Being native to East Asia, natural habitats of goldfish (*Carassius auratus*) are shallow slow-moving rivers, lakes and ponds with dense submerged vegetation. It is historically considered as ornamental fish and is widely used as model organism in biomedical research (Blanco and Unniappan, 2022). Goldfish (*Carassius auratus*) skin has a two-layered structure of superficial epidermidis and deeper dermis rich in collagen, mucous cells and pigment cells, i.e., chromatophores that dictate color. Specialized cells like erythrophores and iridophores imparts to gold, red and metallic hues by carotenoid and pteridine pigments (Matsumoto and Obika, 1968). Such rich pigment composition of goldfish skin may impart to the formation of diverse microbial communities. Therefore, to explore this, we selected goldfish (*Carassius auratus*) for isolation of pigmented bacteria and notably, a tan-colored, melanin-producing organism, namely, *Shewanella xiamenensis*, was isolated from goldfish gills.

*Shewanella xiamenensis* was first reported from coastal sediment in Xiamen, China, in 2010. Typical characteristics include tan or yellowish brown-colored colonies producing melanin pigment (Huang et al., 2010). It is Gram-negative, facultatively anaerobic bacterium recognized for its significant potential in bioremediation, especially for detoxifying heavy metals and industrial effluents (Darma et al., 2022). During this study, *Shewanella xiamenensis* was also investigated for pigment production which further showed similarity with pigment melanin through FTIR studies after comparing with standard FTIR spectra.

Prickles/thorns of the genus *Rosa* are the first line of defense against different biotic and abiotic stresses, specially plays a key role in herbivore defense (Zhou et al., 2021). They are rigid, indigestible and excellent physical barrier that restricts the free movement of nutrients leading to the starvation of pathogens (Doughari, 2015). Therefore, ideally this composition of prickles/thorns are not suitable for growth and development of microorganisms. However chemical composition wise, prickles are the polymers of hemicellulose, cellulose, lignin, mineral substances, suberin etc. (Baroni et al., 2012). Such sugars may support the growth of microorganisms. Therefore, second extreme niche selected in this study was rose prickles/thorns and as a result bright orange-colored endophytic bacteria, *Paracoccus* sp. was successfully isolated from rose prickles/thorns.

This orange-colored Gram-negative coccus was first reported by Harker et al. in 1998 as a contaminant on a nutrient agar plate. The bright orange color of the colony is due to the synthesis of large amounts of carotenoids, i.e., astaxanthin. *Paracoccus* sp. is a promising bacterium for bioremediation, particularly for treating dairy industry wastewater (whey) due to its high β-galactosidase enzyme activity (Kalathinathan and Kodiveri Muthukaliannan, 2021). Moreover *Paracoccus* sp. GSM2 can decolorize up to 800 mg/L of reactive violet 5 dye within 38 h, showing high salt tolerance (up to 2% w/v) and effectiveness in treating textile effluent (Bheemaraddi et al., 2014). Present study also encompasses, isolation of *Paracoccus* sp. (THN1) from unconventional niche, i.e., rose prickles. Pigment characterization through FTIR studies further reported the astaxanthin nature of *Paracoccus* pigment.

*The genus Kocuria, belongs to the group of Actinobacteria and is known for its ability to survive under extreme conditions. It’s diverse environments include soil, marine environments, desserts. Members of the genus Kocuria are Gram positive cocci, documented to produce biotechnologically important metabolites like auxins, pigments, extracellular polysaccharides and antibiotics* (Li et al., 2025). They are known to remove heavy metals like lead and copper via biosorption and degrade hydrocarbons including crude oil and diesel (Heidari et al., 2025). It produces vibrant yellow colored non-diffusible pigment that primarily belong to the carotenoid’s groups, including beta-carotene, lycopene and sarcinaxanthin (Weerasinghe et al., 2025). The present study reported the occurrence of such yellow-colored colony as an incidental laboratory isolate on nutrient agar plate which was later identified as Kocuria sp. (YC). Its pigment characterization through FTIR revealed its beta-carotene nature.

The present study investigated the biodegradation potential of the tested isolates. The *Shewanella xiamenensis* (U2f) demonstrated the visible decolorization and degradation of the Congo red dye at a concentration of 200 mg/mL which were then confirmed by FTIR and HPLC analysis. Literature suggests that the congo red dye degradation is mostly attributed to the involvement of the enzymes like azoreductase which are responsible to cleave the azo bonds (–N = N) present in the Congo red dye (Sankaranarayanan et al., 2021). These findings are also supported by previous report of Silva et al. (2022) where *Shewanella xiamenensis* G5-03 strain was observed to degrade the Congo red with the dye concentration of 50–750 mg L^−1^. They observed the degradation patterns for different parameters like temperature (30–35 °C), pH (10.0), incubation time (10 h), dye concentration (50–750 mg L^−1^) and static condition. In this study they found that *S. xiamenensis* G5-03 might be valuable for the bioremediation of Congo red dye. Another study by Tambe et al. (2026) showed the biodegradation potential of four bacterial strains isolated form textile effluent, *Escherichia* spp.*, Staphylococcus* spp.*, Pseudomonas* spp. *and Enterobacter* spp. and they were evaluated for their decolorization efficiency against the methyl orange and Congo red dyes. After testing the isolates for different parameters, they found that *Escherichia* spp. *and Pseudomonas* spp. exhibited the highest decolorization efficiency at room temperature and pH 9 under static aerobic conditions.

In contrast, the isolates THN1 (*Paracoccus* sp.) and YC (*Kocuria* sp.) successfully decolorized and degraded the malachite green dye at a concentration of 300 mg/mL and 200 mg/mL, respectively. Previous studies reported the involvement of enzyme laccases which is an oxidative enzyme and known to act against the triphenylmethane dyes (Adenan et al., 2021). However detailed studies are necessary to confirm the involvement of such enzymes in specific dye degradation in both the cases. Visible dye degradation of both the isolates was then confirmed by FTIR and HPLC analysis of standard as well as test groups.

Considering the *Paracoccus* sp. in dye degradation, studies conducted by Nisha et al. (2015) showed the biodegradation of dimethylformamide (DMF) by two *Paracoccus* sp. strains MKU1 and MKU2. MKU1 and MKU2 found to degrade 55 and 46% of the initially supplied DMF (1% v/v). A report by Bheemaraddi et al., in 2014 confirmed the decolorization of reactive violet dye 5 up to 800 mg/mL within 38 h. Maurya et al. (2023) performed genomic analysis of metabolically versatile *Paracoccus* sp. strain DMF (P. DMF) known to degrade a recalcitrant organic solvent N, N-dimethylformamide (DMF) which was initially isolated from domestic waste water treatment plant in Kanpur (India). *Paracoccus pantotrophus* has been studied to decolorize the melanoidins from distillery effluents under static conditions. Maximum decolorization was 81% in presence of glucose and NH_4_NO_3_ (Santal et al., 2016). Although *Paracoccus* species has been extensively reported for xenobiotics and dye degradation but current study highlights the potential of *Paracoccus* for malachite green dye degradation (up to 200 mg/mL dye conc. With 90% efficiency).

According to the Parshetti et al. (2006), the strain *Kocuria rosea* MTCC 1532 was found to degrade the malachite green (MG) dye. The bioremediation potential of the strain was tested by TLC, UV-Vi’s spectroscopy, HPLC and FTIR analysis. *K. rosea* showed 100% decolorization of MG (50 mg/L) within 5 h at static anoxic condition whereas there was no decolorization at shaking condition. Decolorization of some other dyes like azo (methyl orange, amido black, Congo red), triphenylmethane (cotton blue, methyl violet, crystal violet) and textile dyes such as monochloro-triazine dyes (reactive blue 25, direct blue-6, reactive yellow 81, reactive red 141, reactive green 19 A) were tested with *K. rosea* (dye conc. 50 mg/L each). Similarly, *Kocuria* sp. (YC) identified as an incidental laboratory isolate during this study was found to decolorize and degrade the malachite green dye (up to 200 mg/mL) with 89% efficiency further confirmed by FTIR analysis.

Moreover, FTIR and HPLC techniques has gained more attention for investigation of dye degradation by microbes. Khan and Joshi (2019) studied the toxic azo dye degradation by *Bacillus pumilus* and *Paenibacillus thiaminolyticus* and confirmation of degradation by FTIR analysis. Similarly, the High-Performance Liquid Chromatography (HPLC) profiles obtained in this study provides definitive chemical confirmation that observed decolorization was driven by active microbial biodegradation rather than simple physical adsorption onto biomass. The complete loss of the original dye peaks and the appearance of new, smaller molecular peaks prove that the bacteria have successfully broke apart the core dye structure. The HPLC profile of dye liquid after the treatment with *Shewanella xiamenensis* (U2f) indicated that the strain successfully broke down the complex Congo red dye. This matches the findings of Selva Raj et al. (2012), where bacterial isolates were used for the degradation of the Congo red dye. The elution profile obtained for the bacteria treated samples significantly differed from the control in terms of number, height of peaks obtained and retention time. The HPLC profile of Congo red treated with bacterial isolate *Hafnia alvei* showed 3 different peaks when compared with the control. Srinivasan and Sadasivam in 2018 demonstrated the similar aerobic-microaerophilic biodegradation of textile azo dyes by *Aeromonas hydrophila* MTCC 1739 and *Lysinibacillus sphaericus* MTCC 9523 and further analysis of degradation by UV-Vi’s spectroscopy, FTIR and HPLC.

Beside individual culture of *Paracoccus* sp. (THN1) and *Kocuria* sp. (YC), co-culture of both the isolates in Zobell Marine broth (ZMB) proved best for decolorization and degradation of malachite green dye at 200 mg/mL conc. Within 16 h under shaking condition with 100% efficiency. The degradation was further confirmed by HPLC analysis. Degradation and decolorization of azo dyes by bacterial-fungal consortium is also reported effectively with FTIR and GC–MS analysis (Henagamage and Peries, 2023). Additionally microbial interaction between yeast and *Pseudomonas* sp. has led the efficient degradation of textile dye rubin G3P which was then confirmed by FTIR and HPLC analysis (Phugare et al., 2011). Mishra et.al. (2020) reported the degradation of mixed dyes like reactive red 21 (RR21) and reactive oxygen 16 (RO16) present in wastewater by *Pseudomonas aeruginosa* 23 N1. The degradation process was analyzed through UV-Vi’s spectroscopy, FTIR and GC–MS techniques. This result suggests the benefits of using of consortium to aid the process of biodegradation at higher dye concentrations. Further it provides the way for process optimization to make the degradation more efficient.

## 5 Conclusion

The study demonstrates the bioremediation potential of the pigmented bacteria isolated from diverse ecological niches like goldfish gills, rose prickles/thorns and an incidental laboratory isolate against the structurally and chemically diverse group of dyes. From goldfish gills, tan colored, melanin producing U2f isolate was identified as *Shewanella xiamenensis*. Rose prickle/thorn endophytic isolate THN1 producing bright orange-colored colonies was identified as *Paracoccus* sp. and it was found to produce a carotenoid pigment astaxanthin. Incidental laboratory isolate YC was identified as *Kocuria* sp. producing yellow colonies with beta – carotene pigment. The chemical composition of pigments was confirmed by Fourier transform infra-red Spectroscopy (FTIR) technique.

The goldfish gill isolate *Shewanella xiamenensis* (U2f) was seen to decolorize and degrade the Congo red dye at the concentration of 200 mg/mL with 70% efficiency and tolerated the dye concentration up to 400 mg/mL. The rose prickle/thorn endophyte, *Paracoccus* sp. (THN1) and incidental laboratory isolate, *Kocuria* sp. (YC) decolorized and degraded the triphenylmethane dye malachite green. Both the isolates were seen to degrade the dye at a concentration of 200 mg/mL with 80–91% efficiency and tolerated the dye concentration up to 500 mg/mL. Moreover consortium of *Paracoccus* sp. (THN1) and *Kocuria* sp.(YC) proved best for malachite green dye degradation within 16 h under shaking conditions with 100% efficiency. The biodegradation of the dyes were confirmed by UV–Vis spectroscopic analysis followed by FTIR spectroscopy and HPLC. Extra absorbance peaks appeared in decolorized solution, probably resulting from the absorbance of metabolites or degraded fragments of dye molecules. These findings suggests the suitability of the isolates for treating the industrial effluent.

## 6 Future prospectives

Future work could focus on optimizing the parameters such as the temperature, pH, nutrient availability in order to maximize the degradation capacity of the isolates. To assess the toxicity of the degradation intermediates and the final products could be of major importance to ensure that the treatment does not produce any harmful compounds. The whole genome studies, could give a valuable insight of the metabolic pathways and genes responsible for the biodegradation of the dyes. Detailed experimental analysis and establishment of relationship between pigment production and dye degradation is also necessary in such type of study. The major aspect of the future study, is to test the efficiency of the isolates against the textile effluents which would also involve the chemical characterization of the effluent in order to know the chemical composition of the wastewater and design the bioremediation strategy. Consortium based biodegradation ability of the isolates would be evaluated in pilot scale then into real world effluent systems. It is also essential to evaluate feasibility, cost-effectiveness of the bioremediation process to determine its practical implementation for large-scale effluent treatment.

## Data Availability

All data has been deposited within the NCBI database with these accession numbers: PZ276692, PX778014, OQ923795.
