# Supplementary material for: Exploration of novel ecological niches for pigmented bacteria with dye bioremediation potential
Source: Front Microbiol. 2026 Oct 1;17:1895442. doi: 10.3389/fmicb.2026.1895442 (PMC13668870; doi:10.3389/fmicb.2026.1895442)
Supplement: Supplementary file 1 [file Supplementary_file_1.docx]

**Supplementary Files**

**Table 1: % decolorization of congo red dye degradation by U2f bacteria (1 to 500 mg/mL)**

| **Conc of dye (mg/ml)** | **Control**  **Abs** | **U2f (test)**  **Abs** | **Δ Absorbance**  **(Control-Test)** | **% decolorization** |
| --- | --- | --- | --- | --- |
| **100** | 4 | 1.274 | 2.726 | **68.1** |
| **200** | 4 | 1.853 | 2.147 | **53.6** |
| 300 | 4 | 4 | 0 | 0 |
| 400 | 4 | 4 | 0 | 0 |
| 500 | 4 | 4 | 0 | 0 |
| 10 | 4 | 0.228 | 3.772 | **94.3** |
| 20 | 4 | 0.226 | 3.774 | **94.4** |
| 30 | 4 | 0.232 | 3.768 | **94.2** |
| 40 | 4 | 0.236 | 3.764 | **94.1** |
| 50 | 4 | 0.254 | 3.746 | **93.6** |
| 1 | 0.589 | 0.112 | 0.477 | **80.9** |
| 2 | 0.865 | 0.129 | 0.736 | **85.0** |
| 3 | 1.098 | 0.139 | 0.959 | **87.3** |
| 4 | 1.352 | 0.146 | 1.206 | **89.2** |
| 5 | 2.021 | 0.159 | 1.862 | **92.1** |

**Table 2: % decolorization of malachite green dye degradation by THN1 bacteria (1 to 500 mg/mL)**

| **Conc of dye (mg/ml)** | **Control**  **Abs** | **THN1 (Test)**  **Abs** | **Δ Absorbance (Control-Test)** | **% decolorization** |
| --- | --- | --- | --- | --- |
| 100 | 0.602 | 0.102 | 0.500 | 83.0 |
| **200** | 1.974 | 0.165 | 1.809 | **91.6** |
| 300 | 2.906 | 1.04 | 1.866 | 64.2 |
| 400 | 3.891 | 3.245 | 0.646 | 16.6 |
| **500** | **3.925** | **3.543** | **0.382** | **9.7** |
| 10 | 0.173 | 0.04 | 0.133 | 76.8 |
| 20 | 0.295 | 0.11 | 0.185 | 62.7 |
| 30 | 0.439 | 0.151 | 0.288 | 65.6 |
| **40** | **0.635** | **0.255** | **0.380** | **59.8** |
| **50** | **0.805** | **0.345** | **0.460** | **57.1** |
| 1 | 0.068 | 0.020 | 0.048 | 70.5 |
| 2 | 0.081 | 0.018 | 0.063 | 77.7 |
| 3 | 0.09 | 0.015 | 0.075 | 83.3 |
| 4 | 0.102 | 0.013 | 0.089 | 87.2 |
| **5** | **0.115** | **0.011** | **0.104** | **90.4** |

**Table 3: % decolorization of malachite green dye degradation by YC bacteria (1 to 500 mg/mL)**

| **Conc. of dye (mg/ml)** | **Control**  **Abs** | **YC(test)**  **Abs** | **Δ Absorbance (Control-Test)** | **% decolorization** |
| --- | --- | --- | --- | --- |
| **100** | **0.623** | **0.108** | **0.515** | **82.6** |
| **200** | **1.974** | **0.211** | **1.763** | **89.3** |
| 300 | 2.906 | 1.883 | 1.023 | 35.2 |
| 400 | 3.891 | 3.577 | 0.314 | 8.0 |
| **500** | **3.925** | **3.689** | **0.236** | **6.0** |
| **10** | **0.256** | **0.072** | **0.184** | **71.8** |
| 20 | 0.326 | 0.09 | 0.236 | 72.3 |
| 30 | 0.439 | 0.12 | 0.319 | 72.6 |
| 40 | 0.635 | 0.238 | 0.397 | 62.5 |
| **50** | **0.805** | **0.212** | **0.593** | **73.6** |
| **1** | **0.068** | **0.051** | **0.017** | **25.0** |
| 2 | 0.081 | 0.063 | 0.018 | 22.2 |
| 3 | 0.090 | 0.072 | 0.018 | 20.0 |
| 4 | 0.102 | 0.085 | 0.017 | 16.6 |
| **5** | **0.115** | **0.096** | **0.019** | **16.5** |

**Table 4 – Signature peaks for congo red dye**

| **Peak cm^-1^** | **Functional Group** |
| --- | --- |
| 3250 to 2980 | OH stretch |
| 1620cm | azo linkage |
| 1598cm | N=N stretching |
| 1096, 1371 | S-O stretching and S=O bond |

**Table 5 – Signature peaks for malachite green dye**

| **Peak cm^-1^** | **Functional Group** |
| --- | --- |
| 1315-1600 | C-N stretching |
| 1590 | C=C Stretching |
| 3200-3500 | OH stretch |

**Statistical Analysis -**

**Table 6 – % Decolorization of congo red dye U2f (*Shewanella xiamenensis*) isolate (1 to 5 mg/mL).**

| Conc.  (mg/mL) | Control | U2f (a) | U2f (b) | U2f  (c ) | %  decolorization (a) | %  decolorization (b) | %  decolorization (c ) | Mean % decolorization | Standard deviation |
| --- | --- | --- | --- | --- | --- | --- | --- | --- | --- |
| 1 | 0.589 | 0.112 | 0.105 | 0.116 | 80.9 | 82.1 | 80.3 | 81.1 | 0.916 |
| 2 | 0.865 | 0.129 | 0.147 | 0.136 | 85 | 83 | 84.2 | 84.06 | 1.006 |
| 3 | 1.098 | 0.139 | 0.114 | 0.109 | 87.3 | 89.6 | 90 | 88.96 | 1.457 |
| 4 | 1.352 | 0.146 | 0.15 | 0.138 | 89.2 | 88.9 | 89.7 | 89.26 | 0.404 |
| 5 | 2.021 | 0.159 | 0.132 | 0.146 | 92.1 | 93.4 | 92.7 | 92.73 | 0.650 |

**Table 7 – % Decolorization of congo red dye U2f (*Shewanella xiamenensis*) isolate (10 to 50 mg/mL).**

| Conc. (mg/mL) | Control | U2f (a) | U2f (b) | U2f (c ) | % decolorization  (a) | %  decolorization  (b) | %  decolorization  (c) | Mean  % decolorization | Standard deviation |
| --- | --- | --- | --- | --- | --- | --- | --- | --- | --- |
| 10 | 4 | 0.228 | 0.345 | 0.271 | 94.3 | 91.3 | 93.3 | 92.96 | 1.527 |
| 20 | 4 | 0.226 | 0.221 | 0.267 | 94.3 | 94.4 | 93.3 | 94.00 | 0.608 |
| 30 | 4 | 0.232 | 0.256 | 0.302 | 94.2 | 93.6 | 92.4 | 93.40 | 0.916 |
| 40 | 4 | 0.236 | 0.245 | 0.278 | 94.1 | 93.8 | 93.1 | 93.66 | 0.513 |
| 50 | 4 | 0.254 | 0.216 | 0.197 | 93.6 | 94.6 | 95.1 | 94.43 | 0.763 |

**Table 8 – % Decolorization of congo red dye U2f (*Shewanella xiamenensis*) isolate (100 to 500 mg/mL).**

| Conc.  (mg/mL) | Control | U2f (a) | U2f (b) | U2f  (c ) | % decolorization (a) | % decolorization(b) | % decolorization (c ) | Mean % decolorization | Standard deviation |
| --- | --- | --- | --- | --- | --- | --- | --- | --- | --- |
| 100 | 4 | 1.274 | 1.098 | 1.134 | 68.1 | 72.5 | 71.6 | 70.73 | 2.324 |
| 200 | 4 | 1.853 | 1.906 | 1.823 | 53.6 | 52.3 | 54.4 | 53.43 | 1.059 |
| 300 | 4 | 4 | 4 | 4 | 0 | 0 | 0 | 0 | 0 |
| 400 | 4 | 4 | 4 | 4 | 0 | 0 | 0 | 0 | 0 |
| 500 | 4 | 4 | 4 | 4 | 0 | 0 | 0 | 0 | 0 |

**Table 9 – % decolorization of malachite green dye by THN1 isolate (*Paracoccus sp.*) (1 to 5 mg/mL)**

| Conc.  (mg/mL) | Control | THN1  (a) | THN1  (b) | THN1  (c) | % decolorization (a) | %  decolorization (b) | %  decolorization (c ) | Mean %  decolorization | Standard deviation |
| --- | --- | --- | --- | --- | --- | --- | --- | --- | --- |
| 1 | 0.068 | 0.02 | 0.024 | 0.023 | 70.5 | 64.7 | 66.1 | 67.10 | 3.026 |
| 2 | 0.081 | 0.018 | 0.019 | 0.017 | 77.7 | 76.5 | 79 | 77.73 | 1.250 |
| 3 | 0.09 | 0.015 | 0.017 | 0.015 | 83.3 | 81.1 | 83.3 | 82.56 | 1.270 |
| 4 | 0.102 | 0.013 | 0.01 | 0.017 | 87.2 | 90.1 | 83.3 | 86.86 | 3.412 |
| 5 | 0.115 | 0.011 | 0.016 | 0.007 | 90.4 | 86 | 93.9 | 90.10 | 3.958 |

**Table 10 - % decolorization of malachite green dye by THN1 isolate (*Paracoccus sp*.) (10 to 50 mg/mL)**

| Conc. (mg/mL) | Control | THN1  (a) | THN1  (b) | THN1  (c) | % decolorization (a) | %  decolorization (b) | %  decolorization  (c) | Mean % decolorization | Standard deviation |
| --- | --- | --- | --- | --- | --- | --- | --- | --- | --- |
| 10 | 0.173 | 0.04 | 0.038 | 0.041 | 76.8 | 78 | 76.3 | 77.03 | 0.873 |
| 20 | 0.295 | 0.11 | 0.117 | 0.108 | 62.7 | 60.3 | 63.3 | 62.10 | 1.587 |
| 30 | 0.439 | 0.151 | 0.155 | 0.149 | 65.6 | 64.6 | 66 | 65.40 | 0.721 |
| 40 | 0.635 | 0.255 | 0.257 | 0.251 | 59.8 | 59.5 | 60.4 | 59.90 | 0.458 |
| 50 | 0.805 | 0.345 | 0.34 | 0.349 | 57.1 | 57.7 | 56.6 | 57.13 | 0.550 |

**Table 11 - % decolorization of malachite green dye by THN1 isolate (*Paracoccus sp*.) (100 to 500 mg/mL)**

`

| Conc.  (mg/mL) | Control | THN1  (a) | THN1  (b) | THN1  (c) | %  decolorization (a) | %  decolorization (b) | %  decolorization (c ) | Mean % decolorization | Standard deviation |
| --- | --- | --- | --- | --- | --- | --- | --- | --- | --- |
| 100 | 0.623 | 0.158 | 0.156 | 0.16 | 74.6 | 74.9 | 74.3 | 74.60 | 0.300 |
| 200 | 1.974 | 0.165 | 0.17 | 0.162 | 91.6 | 91.3 | 91.7 | 91.53 | 0.208 |
| 300 | 2.906 | 1.04 | 1.1 | 1.12 | 64.2 | 62.1 | 61.4 | 62.56 | 1.457 |
| 400 | 3.891 | 3.245 | 3.242 | 3.249 | 16.6 | 16.6 | 16.4 | 16.53 | 0.115 |
| 500 | 3.925 | 3.543 | 3.55 | 3.541 | 9.7 | 9.5 | 9.7 | 9.633 | 0.115 |

**Table 12 - % decolorization of malachite green decolorization by YC (*Kocuria sp.)* isolate (1 to 5 mg/mL)**

| Conc.  (mg/mL) | Control | YC  (a) | YC  (b) | YC  (c ) | %  decolorization (a) | %  decolorization (b) | %  decolorization (c) | Mean % decolorization | Standard deviation |
| --- | --- | --- | --- | --- | --- | --- | --- | --- | --- |
| 1 | 0.068 | 0.025 | 0.023 | 0.027 | 63.2 | 66.1 | 60 | 63.10 | 3.051 |
| 2 | 0.081 | 0.03 | 0.029 | 0.026 | 62.9 | 64.1 | 67.9 | 64.96 | 2.610 |
| 3 | 0.09 | 0.028 | 0.03 | 0.029 | 68.8 | 66.6 | 67.7 | 67.70 | 1.100 |
| 4 | 0.102 | 0.032 | 0.034 | 0.031 | 68.8 | 66.6 | 69.6 | 68.33 | 1.553 |
| 5 | 0.115 | 0.033 | 0.031 | 0.035 | 71.3 | 73 | 69.5 | 71.26 | 1.750 |

**Table 13 - % decolorization of malachite green dye by YC isolate (*Kocuria sp.)* (10 to 50 mg/mL)**

| Conc.  (mg/mL) | Control | YC  (a) | YC  (b) | YC  (c ) | %  decolorization  (a) | %  decolorization (b) | %  decolorization (c) | Mean  %  decolorization | Standard deviation |
| --- | --- | --- | --- | --- | --- | --- | --- | --- | --- |
| 10 | 0.256 | 0.072 | 0.069 | 0.071 | 71.8 | 73 | 72.2 | 72.33 | 0.611 |
| 20 | 0.326 | 0.09 | 0.088 | 0.087 | 72.3 | 73.1 | 73.3 | 72.90 | 0.529 |
| 30 | 0.439 | 0.12 | 0.126 | 0.109 | 71.2 | 71.2 | 75.1 | 72.50 | 2.251 |
| 40 | 0.635 | 0.172 | 0.17 | 0.169 | 73.2 | 73.2 | 73.3 | 73.23 | 0.057 |
| 50 | 0.805 | 0.216 | 0.212 | 0.215 | 73.6 | 73.6 | 73.2 | 73.46 | 0.230 |

**Table 14 - % decolorization of malachite green dye by YC isolate (*Kocuria sp*.) (100 to 500 mg/mL)**

| Conc.  (mg/mL) | Control | YC  (a) | YC  (b) | YC  (c) | % decolorization (a) | % decolorization (b) | % decolorization (c) | Mean  %  decolorization | Standard Deviation |
| --- | --- | --- | --- | --- | --- | --- | --- | --- | --- |
| 100 | 0.623 | 0.108 | 0.105 | 0.107 | 82.6 | 83.1 | 82.2 | 82.63 | 0.450 |
| 200 | 1.974 | 0.211 | 0.217 | 0.203 | 89.3 | 89 | 89.7 | 89.33 | 0.351 |
| 300 | 2.906 | 1.883 | 1.889 | 1.885 | 35.2 | 34.9 | 35.1 | 35.06 | 0.152 |
| 400 | 3.891 | 3.577 | 3.567 | 3.572 | 8 | 8.3 | 8.1 | 08.13 | 0.152 |
| 500 | 3.925 | 3.689 | 3.681 | 3.69 | 6 | 6.2 | 5.9 | 06.03 | 0.152 |
